# Astrocyte–neuron crosstalk through extracellular vesicle-shuttled miRNA-382-5p promotes traumatic brain injury

**DOI:** 10.1038/s12276-024-01355-3

**Published:** 2024-12-02

**Authors:** Qing Hu, Xun Wu, Chengxuan Guo, Tinghao Wang, Hao Guo, Jin Wang, Bodong Wang, Wenxing Cui, Hao Bai, Jinpeng Zhou, Leiyang Li, Liying Han, Liang Cao, Shunnan Ge, Guodong Gao, Ting Wang, Zhenyong Wu, Wei Guo, Yan Qu, Jing Feng, Haixiao Liu

**Affiliations:** 1 Department of Neurosurgery, Tangdu Hospital, Xi’an, Shaanxi China; 2 Shaanxi Clinical Research Center for Neurosurgical Diseases, Xi’an, Shaanxi China; 3 Department of Neurosurgery, Zhejiang Provincial People’s Hospital, Hangzhou, Zhejiang China; 4 Department of Neurosurgery, The 960th Hospital of the PLA Joint Logistics Support Force, Jinan, Shandong China; 5 Department of Traditional Chinese Medicine, Tangdu Hospital, Xi’an, Shaanxi China; 6 State Key Laboratory of Chemical Biology, Shanghai Institute of Materia Medica, Chinese Academy of Science, Shanghai, China; 7 University of Chinese Academy of Sciences, Beijing, China; 8 Shandong Laboratory of Yantai Drug Discovery, Bohai Rim Advanced Research Institute for Drug Discovery, Yantai, Shandong China; 9 Shanghai Institute of Materia Medica, Chinese Academy of Sciences, Shanghai, China; 10 Department of Biomedical Engineering, Fourth Military Medical University, Xi’an, Shaanxi China

**Keywords:** Cell death in the nervous system, Drug delivery

## Abstract

Although astrocytes undergo functional changes in response to brain injury and may be the driving force of subsequent neuronal death, the underlying mechanisms remain incompletely elucidated. Here, we showed that extracellular vesicle (EV)-shuttled miRNA-382-5p may serve as a biomarker for the severity of traumatic brain injury (TBI), as the circulating EV-miRNA-382-5p level was significantly increased in both human patients and TBI model mice. Mechanistically, astrocyte-derived EVs delivered the shuttled miRNA-382-5p to mediate astrocyte–neuron communication, which promoted neuronal mitochondrial dysfunction by inhibiting the expression of optic atrophy-1 (OPA1). Consistent with these findings, genetic ablation of neuronal OPA1 exacerbated mitochondrial damage and neuronal apoptosis in response to TBI. Moreover, engineered RVG-miRNA-382-5p inhibitor-EVs, which can selectively deliver a miRNA-382-5p inhibitor to neurons, significantly attenuated mitochondrial damage and improved neurological function after TBI. Taken together, our data suggest that EV-shuttled miRNA-382-5p may be a critical mediator of astrocyte-induced neurotoxicity under pathological conditions and that targeting miRNA-382-5p-OPA1 signaling has potential for clinical translation in the treatment of traumatic brain injury.

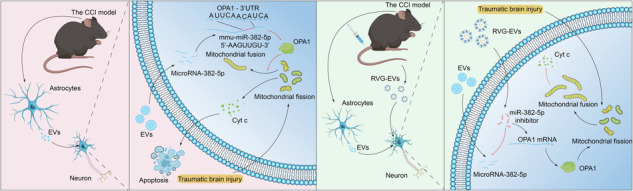

## Extracellular Vesicles Reveal Key Role in Brain Injury

Traumatic brain injury often results in long-term disabilities. This study investigates how astrocytes contribute to nerve cell damage after TBI, focusing on extracellular vesicles and microRNAs, specifically miRNA-382-5p. The research involved blood samples from TBI patients and healthy people, and mouse experiments, to study the effects of astrocyte-derived EVs carrying miRNA-382-5p on nerve cells. The study found that after TBI, astrocytes release EVs with miRNA-382-5p, which disrupts mitochondrial function in nerve cells, causing damage. The study concludes that astrocyte-derived EVs carrying miRNA-382-5p significantly contribute to nerve cell damage after TBI. By inhibiting miRNA-382-5p, such damage can be reduced, offering a new TBI treatment approach. This research enhances our understanding of TBI and offers potential for targeted therapies. Future implications include using miRNA-382-5p as a diagnostic tool or treatment target to improve TBI outcomes. This summary was initially drafted using artificial intelligence, then revised and fact-checked by the author.

## Introduction

Traumatic brain injury is a notable public health issue, placing considerable strain on the health care system^1–3^. The intricate injury mechanism, comprising primary and secondary injuries, poses challenges for accurate assessment and early-stage implementation of appropriate and effective treatment^4^. Primary injury describes the physical damage sustained during a traumatic event, whereas secondary injury typically occurs within minutes to days after the initial insult and involves a detrimental cascade, such as increased astrocyte activities and alterations in neuronal mitochondrial function^5–7^. Although previous studies revealed that a complex intercellular communication network is involved in secondary injury^8^, the underlying cellular and molecular mechanisms remain largely unknown.

Astrocytes provide critical trophic support for neurons, thus playing essential roles in neural development and maintaining synaptic homeostasis^9^. Interestingly, astrocytes undergo functional alterations in response to central nervous system disorders, leading to a transition from a quiescent state known as “resting astrocytes” to an aberrant state referred to as “reactive astrocytes”^10,11^. Given the considerable heterogeneity of activated astrocytes across various diseases, disease progression stages, and anatomical locations, they can exert diverse effects on neurons, including neuroprotection, neurotoxicity, and facilitation of neural repair. For example, the transfer of functional mitochondria from astrocytes to neurons, facilitated by a calcium-dependent mechanism, represents intercellular crosstalk that contributes to neuroprotection and recovery following stroke^12^. Specialized reactive astrocytes possess the capacity to release neurotoxic substances that can lead to neuronal cell death. For example, the saturated lipids in Apolipoprotein E (APOE) and Apolipoprotein J (APOJ) lipoparticles mediate astrocyte-induced neurotoxicity in neurodegenerative disease^13^. Moreover, during secondary injury following TBI, excessive astrocyte-mediated release of excitatory amino acids such as glutamate and aspartate induces reactive oxygen species (ROS) generation, excitotoxicity and calcium overload, ultimately causing mitochondrial dysfunction and axonal damage^14^. Therefore, exploring the underlying mechanisms by which reactive astrocytes contribute to neuronal death after brain injury with the aim of establishing effective strategies for mitigating secondary injury is imperative.

Extracellular vesicles (EVs) are cell-derived nanovesicles with bilayer lipid membranes that mediate intercellular communication in many homeostatic and pathological processes^15–17^. The biological molecules contained within EVs, such as nucleic acids, lipids, and proteins, can be transferred to recipient cells, leading to the exchange of biological information and the reprogramming of cellular functions^18,19^. Specifically, a class of noncoding RNA molecules typically composed of 19–24 nucleotides can selectively bind to specific messenger RNAs, thereby initiating transcriptional degradation and/or impeding translation^20^. Accumulating evidence supports the effects of EV-mediated microRNA (miRNA) transfer on blood‒brain barrier permeability^21^, neurodegeneration^22^, and axonal remodeling^23^ in the central nervous system (CNS). For example, exosomal miR-342-5p inhibited hypoxia/reoxygenation-induced cardiomyocyte apoptosis by targeting Caspase 9 and Jnk2^24^. Moreover, exosomal miRNA-4315 inhibits tumor cell apoptosis by downregulating the proapoptotic protein Bim^25^. However, the role of exosomal miRNAs in neuronal apoptosis after TBI has not been fully elucidated.

In this study, we showed that EV-miRNA-382-5p levels are significantly increased in both human and mouse samples and can be used as a potential biomarker for predicting TBI outcomes. Astrocyte-derived EV-miRNA-382-5p contributes to neuronal damage by modulating OPA1 signaling, indicating that it is a promising therapeutic target for TBI treatment. In support of this hypothesis, engineered EVs, which can selectively deliver a miRNA-382-5p inhibitor to neurons, significantly alleviate TBI-induced neuronal death and motor deficits in mice. Collectively, our data reveal the role of miRNA-382-5p-OPA1 signaling in the pathogenesis of neuronal death, revealing the translational potential of targeting astrocyte-secreted EVs in brain injury.

## Materials and methods

### Study population

Blood samples were collected from patients with craniocerebral injury and healthy blood donors at Tangdu Hospital of Air Force Medical University, and written informed consent was obtained. During the screening phase, a total of 12 healthy controls and 12 patients with TBI were selected to identify the differentially expressed EV-derived miRNAs after TBI. For further analysis, a total of 60 healthy controls and 60 patients with TBI were included in the validation cohort. All diagnoses were confirmed by two trained neurologists. The participants were adult TBI patients admitted to the emergency room within 24 h after injury; those with a history of CNS disorders, alcohol or drug addiction, impairment of other organs, or language dysfunction were excluded. All potentially eligible patients were assessed by two researchers. Upon admission, a venous blood sample of 10 ml was obtained. Demographic data, the injury mechanism, clinical features, head CT scan findings, and laboratory values were recorded for further analysis. The primary outcome was neurological function after 6 months, as assessed by the GOS score. As mentioned above, patients with GOS scores ranging from 1 to 3 were regarded as having a poor functional outcome, and those with scores ranging from 4 to 5 were regarded as having a good functional outcome. Supplementary Tables 1 and 2 provide an overview of the patients’ characteristics. The study protocol was approved by the Ethics Committee of Tangdu Hospital, the Air Force Military Medical University (approval ID: 202107-19).

### TBI animal model

Consistent with most previous experimental TBI studies^26,27^, our study focused on male mice to eliminate the potential impact of the neuroprotective effects of female sex hormones on the results. Eight-week-old male C57BL/6 mice weighing between 25 and 30 g were utilized in this study. The laboratory procedures were reviewed and approved by the Animal Experiment Center of Air Force Military Medical University (approval ID: IACUC-20210554) and performed in accordance with the Use and Care of Laboratory Animals, and the results are reported in compliance with the ARRIVE guidelines. Neuron-specific OPA1 knockout mice were generated by crossing *Opa1*^f/f^ mice (Cyagen, USA, Inc., CA, USA) with Map2-CreERT2 mice (Shanghai Model Center) and then treating the offspring with 75 mg/kg tamoxifen (#S1238, Selleck) for 5 days.

An in vivo controlled cortical impact (CCI) model of TBI was employed. Briefly, sodium pentobarbital (1%; 50 mg/kg body weight) was administered intraperitoneally for anesthesia induction. A craniotomy was performed on the right side of the skull between the pons and lambda. The mice underwent moderate unilateral controlled cortical impact injury using a baroshock device (RWD Life Sciences, Shenzhen, China); the diameter of the tip was 3.5 mm, the depth was 1.8 mm, the velocity was 2.5 m/s, and the dwell time was 500 ms. In the sham group, a craniotomy without any further intervention was performed. After CCI or the sham operation, the mice were returned to their cages and monitored.

### MiRNA‑382-5p translocation analysis

Astrocytes were transfected with cyanine 3-labeled miRNA-382-5p (Cy3-miR-382-5p) or miRNA mimic NC (GenePharma Inc., Shanghai, China) at a final concentration of 100 nM using Lipofectamine RNAiMAX (Thermo Fisher, #13778150) for 24 h according to the manufacturer’s protocol. ADEVs were cocultured with primary neurons for 24 h after ultracentrifugation.

Subsequently, immunofluorescence staining of primary neurons was conducted. After the primary neurons were blocked with 5% bovine serum albumin (BSA) for 1 h, they were incubated overnight at 4 °C with an anti-Map2 antibody (diluted to 1:200; Abcam, AB254143). The neurons were subsequently washed three times and incubated in the dark at room temperature for 1 h with an Alexa Fluor 488-conjugated goat anti-mouse IgG antibody (diluted to 1:800, Invitrogen, A-11001), after which the nuclei were stained with DAPI. Fluorescence images were captured using a confocal laser scanning microscope (CLSM) (Nikon, A1Si, Japan) and analyzed using ImageJ software. The expression of miRNA-382-5p in primary neurons was assessed using qPCR.

Then, the ADEVs were pretreated with 0.5% Triton X-100 (Sigma‒Aldrich, #T8787) for 5 min and treated with 5 μl of RNase (Thermo Fisher Scientific, #AM2271)^28,29^ for 1 h, followed by the removal of residual Triton X-100 and RNase through washes with PBS and ultracentrifugation at 4 °C for 70 min at a speed of 100,000×g. The expression of miRNA-382-5p in primary neurons was evaluated using qPCR. The miRNA and primer sequences are listed in Supplementary Tables 4 and 5 in the Supplementary Materials.

### Isolation and characterization of EVs

The collected blood samples were immediately placed in a tube precoated with EDTA (Cat# 365974, BD Biosciences) and gently inverted 10 times to mix properly. The plasma was obtained by centrifugation at 1600×*g* for 10 min at 4 °C, and the supernatant was transferred to RNase/DNase-free tubes and stored at −80 °C for further processing. EVs were isolated by ultracentrifugation. The supernatant samples were centrifuged at 10,000×*g* for 30 min at 4 °C to isolate cells and platelets and then centrifuged twice at 100,000×*g* for 70 min at 4 °C with an SW40Ti rotor. The isolated EVs were resuspended in phosphate-buffered saline for further experiments.

EVs were isolated from serum-free Opti-MEM after 24 h of incubation. Following the addition of protease inhibitors to the collected conditioned medium, sequential centrifugation was performed at 300×*g* at 4 °C for 5 min, followed by 2000×*g* at 4 °C for 10 min and 10,000×*g* at 4 °C for 30 min. The obtained supernatants were filtered through a 0.22 μm strainer (Millipore Sigma, Billerica, MA, USA) to remove dead cells and large particles. The supernatants were subsequently subjected to ultracentrifugation at 100,000×*g* for 70 min. After the supernatants were discarded, the EVs were temporarily stored at 4 °C until use in subsequent experiments.

Brain-derived EVs were collected from cortical and hippocampal tissues in the injured area of mice as described previously, with some modifications^30^. Briefly, approximately 30 mg tissue blocks were collected and incubated with 75 U/ml collagenase type 3 (8 μl/mg tissue) for digestion at 37 °C for 20 min. The test tubes were gently inverted every 5 min and immediately placed on ice, after which 1x protease and phosphatase inhibitors were added. The isolated tissue mixture was subsequently centrifuged at 300×*g* at 4 °C for 5 min, 2000×*g* at 4 °C for 10 min, and 10,000×*g* at 4 °C for 30 min to remove cell debris. The supernatant was collected and filtered through a 0.22 μm strainer to eliminate dead cells and large particles. EVs were then harvested via ultracentrifugation at 180,000×*g* using an SW40Ti tube, and the supernatant was subsequently centrifuged for 3 h on a triple sucrose density gradient (0.6 M, 1.3 M, and 2.5 M). After centrifugation, three fractions, F1, F2 and F3, were collected and individually diluted with PBS. Particles from F2 were used in all further experiments. The samples were then centrifuged at 100,000×*g* for 70 min at 4 °C. For morphological observations, the isolated EVs were prepared for TEM imaging. For size and concentration analyses, the EVs were diluted in filtered PBS and examined with a Zetaview instrument (Particle Metrix, Germany). The expression of protein markers of EVs and negative control markers was examined by Western blotting (WB).

### Construction and sequencing of miRNA libraries

Total RNA was extracted from EVs with TRIzol LS reagent (Invitrogen, USA) according to the manufacturer’s protocol. Subsequently, 18–30 nt RNA molecules were enriched through polyacrylamide gel electrophoresis (PAGE). The ligation products were amplified via polymerase chain reaction (PCR) and then reverse-transcribed to produce 140–160 bp DNA libraries. The samples were sequenced on the Illumina NextSeq 500 platform by Gen Denovo Biotechnology Co., Ltd. (Guangzhou, China). Clean reads were acquired by filtering out low-quality reads and trimming adaptor sequences. Unannotated tags were matched to the reference genome using the genomic locations and hairpin structures predicted using Mireap v0.2, and the values of the resulting candidate miRNAs were converted to transcripts per million (TPM) values. The clean tags were compared with the miRBase dataset to identify known miRNAs. miRNAs with a |log2fc| ≥ 1-fold and a *P* value < 0.05 were identified as significantly differentially expressed (DE) miRNAs. A normalized count >4 TPM was considered indicative of maximum reliability.

### Quantitative real-time PCR

Total RNA was isolated from EVs using TRIzol LS reagent (Invitrogen, USA). For the mRNA analysis, the extracted total RNA was reverse transcribed into complementary DNA (cDNA) using a PrimeScript RT reagent kit (Takara). For the analysis of miRNA expression, before the chloroform step of RNA extraction, cel-miR-39 was introduced to minimize the degree of technical variability between samples. The RNA concentrations were confirmed using a NanoDrop spectrophotometer (Thermo Scientific, USA). Reverse transcription of the isolated RNA was performed with the Mir-XTM miRNA First-Stand Synthesis Kit (Takara). RT‒PCR was conducted using a Bio-Rad RT‒PCR system (USA) and a SYBR Premix Ex Taq^TM^ II Kit (Takara). The miRNA and mRNA levels were normalized to those of the endogenous controls U6/cel-miR-39 and β-actin, respectively. The sequences of the primers are listed in Supplementary Table 5. The expression of individual genes was evaluated using the 2^−△△Ct^ method.

### Cell culture and transfection

The 293 T cell line was obtained from GeneChem (Shanghai, China). 293T cells were cultured in Dulbecco’s modified Eagle’s medium (DMEM) supplemented with 10% FBS and 1% mixed antibiotic solution (streptomycin and penicillin) and maintained at 37 °C with 5% CO_2_. Primary neurons were isolated from C57BL/6 mouse embryos as described previously^31^. Briefly, the primary cerebral cortex was rapidly dissected, the meninges were removed, and the tissue was digested with a 0.25% trypsin solution for 10 min at room temperature. Pooled cells were cultured in DMEM/F12 containing 10% FBS, 100 U/ml penicillin, and 100 mg/ml streptomycin for 4 h at 37 °C. A neural matrix medium consisting of B27 supplement (2%), double antibiotic solution (1%), and L-glutamine (1%) was used as the culture medium. The cells were incubated at 37 °C in a controlled atmosphere of 5% CO_2_, and the medium was replaced every three days. Primary neurons were cultured for approximately 7 days before the subsequent experiments.

Primary microglia and astrocytes were isolated from neonatal C57BL/6 mice aged 1–2 days. Briefly, the brains were minced and treated with 0.1% trypsin (Sigma‒Aldrich) and 0.25% DNase (Roche) for 10 min to obtain a cell suspension, which was passed through a 70 µm cell filter. Mixed cultures of glial cells were cultured in DMEM/F12 (containing 10% FBS and 1% double antibiotic solution) until the cells reached confluence. After ten days, the astrocyte monolayer was shaken at 220 rpm for 40 min to remove microglia, leaving only astrocytes in the flask. For subsequent experiments, both astrocytes and microglia were cultured in a 5% CO_2_ atmosphere at 37 °C.

A scratch wound model was established using a 10 μl pipette tip to create linear scratches in the cell layer, with approximately 4 mm between each scratch. The control group did not receive any intervention. Once the primary neurons reached 70% confluence, they were transfected with 100 nM miRNA-382-5p mimic (sense, 5’-GAAGUUGUUCGUGGUGGAUUCG-3’ and antisense, 5’-AAUCCACCACGAACAACUUCUU-3’) or miRNA mimic NC (sense, 5’-UUCUCCGAACGUGUCACGUTT-3’ and antisense, 5’-ACGUGACACGUUCGGAGAATT-3’) (GenePharma Inc., Shanghai, China) using Lipofectamine RNAiMAX (Thermo Fisher, #13778150) for 24 h, according to the manufacturer’s guidelines. A GV314 adenoviral vector expressing OPA1 (Ad-OPA1) and a negative control virus (CON267 CMV-MCS-3FLAG-SV40-EGFP, Ad-Ctrl) were constructed by GeneChem (Shanghai, China). When the cells reached 70% confluence, Ad-OPA1 or Ad-Ctrl was transfected into the cells at an optimal multiplicity of infection (MOI = 9). For further analysis, the transfected cells were harvested at the indicated times.

### Stereotaxic injection of adeno-associated viruses

AAV9-Smpd3-RNAi (AAV-SMPD3-shRNA) or AAV9CON537 (AAV-NC) and AAV5-mmiR382-5p sponge (AAV-miRNA-382-5pi) or AAV5CON517 (AAV-Ctrl) from GeneChem (Shanghai, China) were used for stereotaxic injections. The mice were anesthetized with 1% sodium pentobarbital and placed in a stereotaxic frame for virus injection. The virus was injected into the right cortex (ipsilateral to the injury site) at the following coordinates: 1.80 mm anteroposterior, 2.50 mm mediolateral, and 1.00 mm dorsoventral. A total of 1.5 μl of a 1 × 10^9^ TU/ml AAV suspension was injected at a rate of 0.2 μl/min. The needle was withdrawn over approximately 10 min. The animals were subjected to brain trauma at 14 days after the lentivirus injection. Fluorescence microscopy was used to visualize the cortices and evaluate the efficiency of AAV infection. The number of astrocytes infected with AAV within eight randomly selected grids surrounding the injection area in each cortex, measuring 250 × 500 μm, was subsequently quantified.

### Cell viability and cytotoxicity assays

An MTT Cell Proliferation and Cytotoxicity Assay Kit (Beyotime Biotechnology, Suzhou, China) was employed to assess cell viability in accordance with the manufacturer’s protocol. A lactate dehydrogenase (LDH) release test kit (Abcam, Cambridge, UK) was used to assess cytotoxicity according to the manufacturer’s instructions.

### Luciferase reporter assay

A dual luciferase assay was used to evaluate the binding of miRNA-382-5p to its target gene OPA1. Briefly, 293 cells were seeded in a 96-well plate, grown until they reached 50% confluence, and transfected after 24 h with the miRNA-382-5p mimic (50 nM) or control mimic (50 nM), along with 0.2 μg of psiCheck-OPA1 WT or psiCheck-OPA1 MUT reporter vector, using Lipofectamine 2000 (Invitrogen). After 48 h, the cells were harvested, and luciferase activity was measured using a dual luciferase reporter assay kit (Promega, Weison, USA). The results of 3 independent experiments were analyzed, and each experiment was performed in sextuplicate.

### EV labeling and tracking in vivo

Mock-EVs and RVG-EVs were incubated with DiR (Umibio, Shanghai, China) for 30 min at RT and centrifuged at 100000×*g* for 70 min at 4 °C to remove the free dye. The labeled EVs (200 μg) were subsequently administered intravenously into the mice through the tail vein. EV localization in the whole body and individual organs was assessed with an IVIS imaging system (Xenogen, USA) after 24 h. For the analysis of tissue sections, 1 μM PKH26 (Umibio, Shanghai, China) was incubated with Mock-EVs and RVG-EVs for 30 min at room temperature. After sequential centrifugation as described above, PKH26-labeled EVs were administered to the mice. Fresh brain tissue was subsequently collected, washed, and embedded in optimal cutting temperature medium. After being blocked with 5% bovine serum albumin (BSA) for 1 h, the tissue sections were incubated with an anti-NeuN antibody (1:100 dilution; Millipore, ABN90) overnight at 4 °C. The sections were incubated with an Alexa Fluor 488-conjugated goat anti-guinea pig IgG antibody (Invitrogen, 1:800) at room temperature for 1 h in the dark after being washed three times, followed by nuclear staining with DAPI. Fluorescence images captured with a confocal laser scanning microscope (CLSM) (Nikon, A1Si, Japan) were analyzed using ImageJ.

### Measurement of the cerebral lesion volume and edema

A 3 T animal small MRI scanner (Bruker MRI GmbH, Germany) was used to image mouse brains 1 day following TBI after the mice were anesthetized. Images of the lesion were acquired using an MRI scanner equipped with a radiofrequency surface and a phased array mouse brain coil. The brain tissues were scanned and cut into equidistant coronal sections, each measuring 0.7 mm in thickness. The cerebral lesion volume was assessed by exporting MR images in DICOM format and analyzing them via ImageJ.

The brain water content was evaluated using the wet‒dry weight technique 24 h after TBI induction. The weights of the samples were quickly determined using an analytical scale immediately and after drying at 60 °C for 72 h. The following formula was used to evaluate the brain water content: [(wet weight‒dry weight)/wet weight] × 100%.

### Neurobehavioral analysis

The neurobehavioral analysis was performed one day prior to traumatic brain injury (TBI) and was subsequently repeated on Days 1, 3, 7, and 14 following the surgical procedure. Neurologic deficits were evaluated using modified neurological severity scores (mNSSs), the corner turn test, and the wire hanging test. mNSSs include motor, sensory, balance, and reflex scores and range from 0–18 (0, no neurological impairment; 18, maximum neurological impairment). In the corner turn test, the mice were allowed to enter a 30° curve while exiting a corner by turning either left or right. The direction selected over 10 trials was recorded, and the proportion of left turns was calculated. For the wire hanging assay, a metal wire (1 mm × 55 cm) was horizontally suspended between two posts positioned at a height of 50 cm above ground level, with a pillow placed underneath. The mice were directed to grip the wire using their forelimbs, while their hindlimbs were immobilized with medical adhesive tape. Once the mice were appropriately suspended, they were allowed to hang from the wire. The latency to fall was subsequently measured; any trial in which the latency was <10 s was excluded from the analysis.

### Measurement of reactive oxygen species levels

ROS levels were measured as described previously^32^ by DHE (Yesen) staining. Briefly, mouse brain tissues were collected and frozen at −80 °C for 20 min. They were then sectioned into 15 mm thick slices and incubated with DHE (10 mol/L) for half an hour. Finally, after an examination of the slices by CLSM, the relative ROS fluorescence levels were measured via ImageJ. For the measurement of MDA and MnSOD levels, mouse tissues and cells were collected 24 h after injury and subsequently homogenized in lysis buffer (Beyotime, China) on ice for 30 min. After centrifugation at 4 °C for 10 min at a speed of 12000 rpm, the supernatants were collected. Commercial kits were used to measure MDA (Beyotime, Jiangsu, China) and MnSOD (Beyotime, Jiangsu, China) levels.

### TUNEL assay

The assessment of cell apoptosis was conducted using an In Situ cell death detection kit (TMR Red, Roche). The tissue slices were fixed with 4% paraformaldehyde (PFA) for 2 h and permeabilized with 0.5% Triton X-100 in PBS for 30 min. Then, the slices were stained with TUNEL solution at 37 °C for 1 h and then stained with DAPI solution at 37 °C for 10 min. The degree of apoptosis was determined by calculating the ratio of TUNEL-positive cells to DAPI-stained cells.

### Flow cytometry

Neurons were stained with Annexin-V and propidium iodide (PI) using an Annexin-V-FITC kit (BD Biosciences). The cells were then digested into single-cell suspensions, washed twice with ice-cold PBS, and resuspended in binding buffer at a concentration of 1 × 10^5^ cells/100 μl. After an incubation for 10 minutes in the dark at room temperature, a mixture of 5 μl of Annexin V-FITC and 5 μl of PI was added to the cells. Subsequently, 400 μl of 1× binding buffer (BD Accuri, BD Biosciences’ C6 Plus) was added to each sample, followed by flow cytometry analysis within 1 h.

### Western blotting

The brain tissues and cultured cells were sonicated for 20 minutes, followed by homogenization in ice-cold lysis buffer containing protease inhibitors and phosphatase inhibitors. The samples were subsequently resolved on 10%-15% SDS gels and transferred onto polyvinylidene difluoride (PVDF) membranes (Millipore, USA). After being blocked with 5% BSA for 1 h at room temperature, the membranes were incubated overnight at 4 °C with rabbit anti-CD81 (27855-1-AP, Proteintech), anti-CD9 (20597-1-AP, Proteintech), anti-TSG101 (14497-1-AP, Proteintech), anti-OPA1 (80471, Cell Signaling Technology), anti-Cytochrome c (#4280S, Cell Signaling Technology), anti-Lamp2 (27823-1-AP, Proteintech Group), anti-GM130 (PA5-95727, Invitrogen), anti-VDAC (#4661, Cell Signaling Technology) or anti-β-actin (20536-1-AP, Proteintech) antibodies, followed by an incubation with the corresponding enzyme-linked secondary antibodies (AS003, AS014, ABclonal) for 2 h at room temperature. The protein bands visualized using the Bio-Rad imaging system were analyzed with ImageJ.

### Transmission electron microscopy

The samples were prepared according to a previously described protocol^33^. The mice were anesthetized and perfused using a standard procedure 24 h after injury. The brains were placed in cool Hanks’ balanced salt solution upon removal and then sectioned into 1 mm thick slices along the coronal plane. The cortical samples (1 × 1 × 2 mm^3^) were immersed in 4% glutaraldehyde, incubated at 4 °C overnight, fixed with 1% cesium trioxide for 1 h, dehydrated in a graded series of ethanol solutions, and embedded in resin. The embedded sample blocks were trimmed and sectioned with an ultrathin microtome. The sections were subsequently placed on a 200-slot grid coated with an oval film and imaged using a JEM-1400 electron microscope (JEOL, Tokyo, Japan) and a charge-coupled device camera (OLYPUS, Tokyo, Japan).

### Assessments of mitochondrial morphology and function

Primary neurons were stained with 10 nM MitoTracker Red (40743ES50, Yeasen) for 30 min. CLSM was employed to visualize the mitochondria. ImageJ was used to analyze the mitochondrial length in each group, and the results are presented as the aspect ratio. Mitochondrial ROS levels were assessed using the Mitochondrial™ Red (Invitrogen) assay. Following a 30 min incubation with MitoSOX (5 nmol/L), primary neurons were rinsed twice with warm HBSS. The CLSM-acquired fluorescence images were analyzed with ImageJ software. The transmembrane potential was assessed using the Mitochondrial Membrane Potential Assay Kit with JC-1 (Beyotime, China). Following a 20 min incubation in DMEM containing 10 μM JC-1 at 37 °C, the primary neurons were rinsed twice with PBS. The fluorescence microscopy images were analyzed using ImageJ.

### Loading of RVG-EVs with a miRNA-382-5p inhibitor and intravenous injection

HEK293T cells were plated in 225 cm^2^ flasks (431082, Corning) and cultured at 37 °C with 5% carbon dioxide. When the cells reached approximately 70% to 80% confluence, they were transfected with the RVG-Lamp2b plasmid (71294, Addgene) using Lipofectamine 2000 according to the manufacturer’s guidelines. The medium was changed to EV-free medium at 24 h after transfection. The conditioned medium was collected, and RVG-EVs were isolated from the supernatant of HEK293T cells transfected with the RVG-Lamp2b plasmid through serial centrifugation. As controls, simulated EVs (Mock-EVs) were isolated from untransfected HEK293T cells. The serial centrifugation protocol used for EV collection was the same as that used for extracting EVs from primary astrocyte supernatants. After the removal of the supernatants, the EVs were temporarily stored at 4 °C until use in subsequent experiments.

RVG-EVs were electroporated with an miRNA (miRNA-382-5p inhibitor or control) (GenePharma Inc., Shanghai, China) at a voltage of 350 V/150 mF in a 0.4 cm electroporation cassette. The ratio of RVG-EVs to the inhibitor was approximately 400 μg of RVG-EVs/1 OD of inhibitor. The sequences of the inhibitors are listed in Supplementary Table 4 in the Supplementary Materials. After electroporation, the unloaded inhibitor attached to the surface of the EVs was removed with RNase, and then, the EVs were isolated via serial centrifugation. Mice subjected to brain damage received intravenous injections of either RVG-miRNA-382-5pi-EVs or RVG-NC-EVs starting 1 h after injury and continuing every other day for 12 days.

### Statistical analysis

Categorical and numeric variables were analyzed via descriptive statistics. Categorical data are presented as numbers and percentages. Continuous variables are presented as either the means (standard deviations) or medians (interquartile ranges). Two-group comparisons were conducted using Student’s *t* test, the *χ*^2^ test, or the Mann‒Whitney U test, whereas multiple group comparisons were performed by one-way or two-way ANOVA followed by Tukey’s post hoc test (GraphPad Prism 8.0). Multivariate logistic regression was employed for multivariate analyses. The ORs, together with their 95% confidence intervals (CIs), were also calculated. Statistical analyses were performed using SPSS 24.0 software (IBM Corp., Armonk, NY, USA). *p* < 0.05 was considered to indicate statistical significance.

## Results

### Upregulation of EV-miRNA-382-5p in patients and mice following TBI

**Fig. 1:**
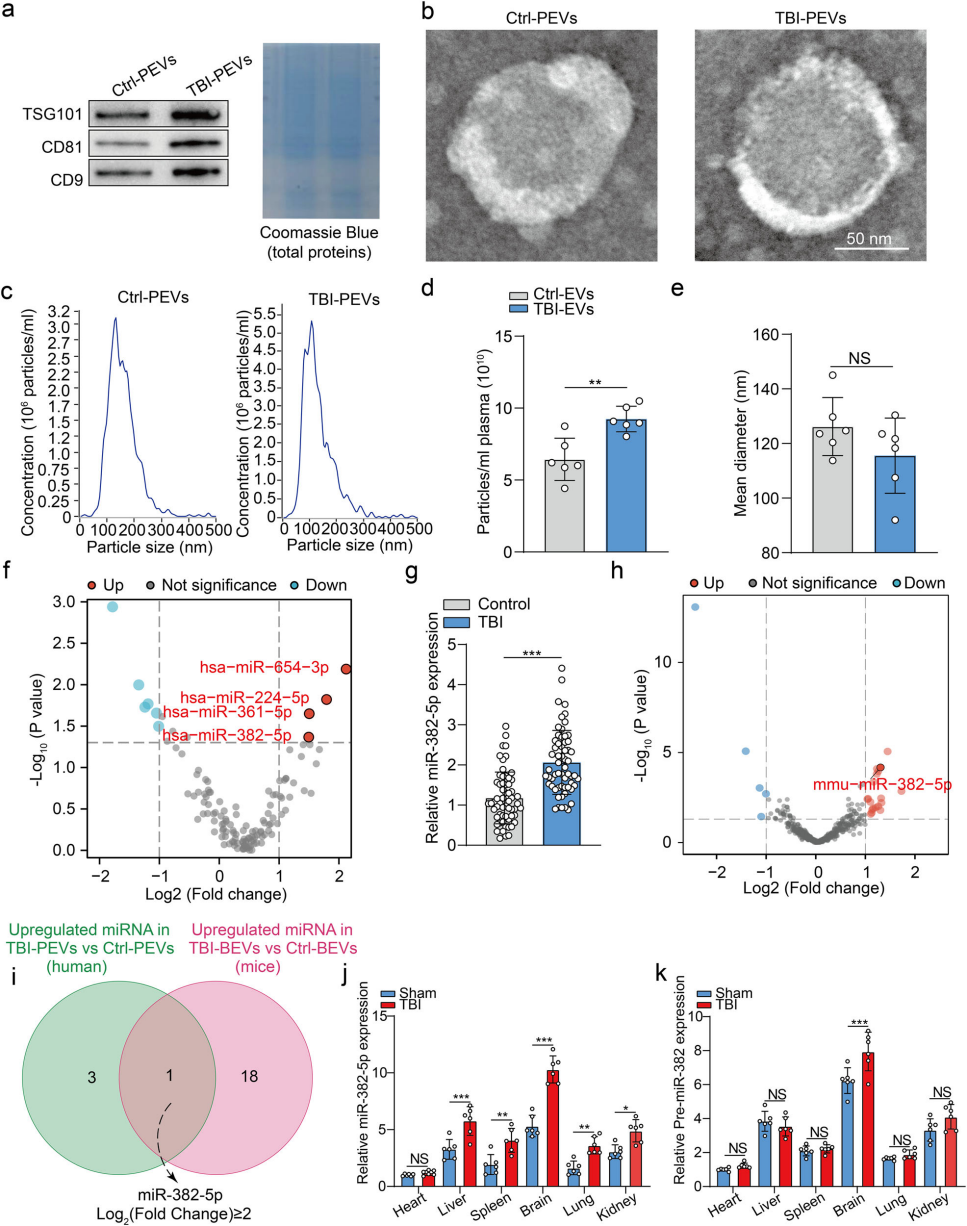
TBI upregulates EV-miRNA-382-5p in patients and mice. **a** Representative Western blot (WB) and image of Coomassie brilliant blue staining (loading control) of EVs isolated from the plasma of TBI patients and normal controls. **b** Representative electron micrograph of isolated EVs. Scale bar, 50 nm. **c** The size distribution and number of particles per milliliter in samples isolated from plasma-derived EVs are shown. **d**, **e** The concentration and size of plasma-derived EVs isolated under the experimental conditions are shown (*n* = 6 per group; Student’s t test). **f** Volcano plots showing the differential expression of miRNAs in plasma EVs from TBI patients and healthy controls (TBI = 12, CON = 12; |log2FC| ≥ 1 and *p* < 0.05). **g** Related fold changes in the mRNA levels of miRNA-382-5p in plasma EVs between TBI patients and healthy controls (*n* = 60 per group; Student’s t test). **h** Volcano plots showing the differential expression of miRNAs in the brain-derived EVs of mice in the TBI group compared with those in the sham group (TBI group=6, sham group=6; |log2FC | ≥1 and *p* < 0.05). **i** Venn diagram showing that miRNA-382-5p was the only significantly upregulated miRNA between the human and mouse groups. **j**, **k** Related fold changes in the expression of miRNA-382-5p and premiR-382 in each organ from mice in the sham and TBI groups (*n* = 6 per group; Student’s *t* test). The data are presented as the means ± SDs; *p < 0.05, **p < 0.01, ***p < 0.001, and NS not significant.

EVs play an important role in regulating the biological functions of recipient cells by delivering biomolecular cargo, such as miRNAs^34–37^. We first isolated plasma-circulating EVs from TBI patients (TBI-PEVs) and age-matched healthy volunteers (Ctrl-PEVs) to investigate the potential roles of EV-shuttled miRNAs in TBI. The quality and purity of the EVs were assessed by Western blotting (WB), transmission electron microscopy (TEM), and nanoparticle tracking analysis (NTA) (Fig. 1a–c). The presence of EV-specific markers (TSG101, CD81, and CD9) was confirmed by WB (Fig. 1a and Supplementary Fig. 1a–c), and TEM revealed that the spherical EVs had a clear typical bilayer with a diameter of <150 nm (Fig. 1b), which was consistent with the NTA results showing that the diameters of the vesicles were mainly in the range of 50–150 nm (Fig. 1c). Interestingly, a distinctive increase in the concentration of circulatory EVs was observed in TBI patients compared with healthy controls (Fig. 1d). After a comparative analysis of the mean diameters of EVs, we observed no statistically significant difference in particle size between the EVs isolated from the plasma of TBI patients and healthy controls, although a trend toward a smaller particle size of the EVs was detected in TBI patients (Fig. 1e). We performed miRNA sequencing to determine the miRNA expression profiles of the circulating EVs and identified various miRNAs in both groups. Strikingly, miRNA-382-5p, miRNA-361-5p, miRNA-224-5p, and miRNA-654-3p were significantly upregulated in TBI patients compared with healthy controls (Fig. 1f). Although these data were further supported by sequencing data from an expanded cohort (Fig. 1g and Supplementary Fig. 2a–c), we could not confirm whether these miRNAs were specifically enriched in injured brain tissue, and the increase in their levels in serum EVs may only indirectly support a potential role for these miRNAs in TBI. To overcome this, we employed a well-established TBI mouse model of controlled cortical impact in vivo to address this limitation, and EV samples were collected and subjected to miRNA sequencing. As shown in Fig. 1h, 18 miRNAs were upregulated in the EVs from injured brain tissue (TBI-BEVs) compared with the EVs from sham tissue (Ctrl-BEVs). Notably, miRNA-382-5p was the only miRNA upregulated in both species (Fig. 1i). Although miRNA-382-5p and its precursor-miR-382 (premiR-382) are expressed in various organs, we detected the significant upregulation of mature miRNA-382-5p and premiR-382 in the brain of the TBI group compared with the sham group (Fig. 1j, k), suggesting a potential pathological role of EV-miRNA-382-5p in both humans and mice.

### Internalization of astrocyte-derived EV-derived miRNA-382-5p by primary neurons

**Fig. 2:**
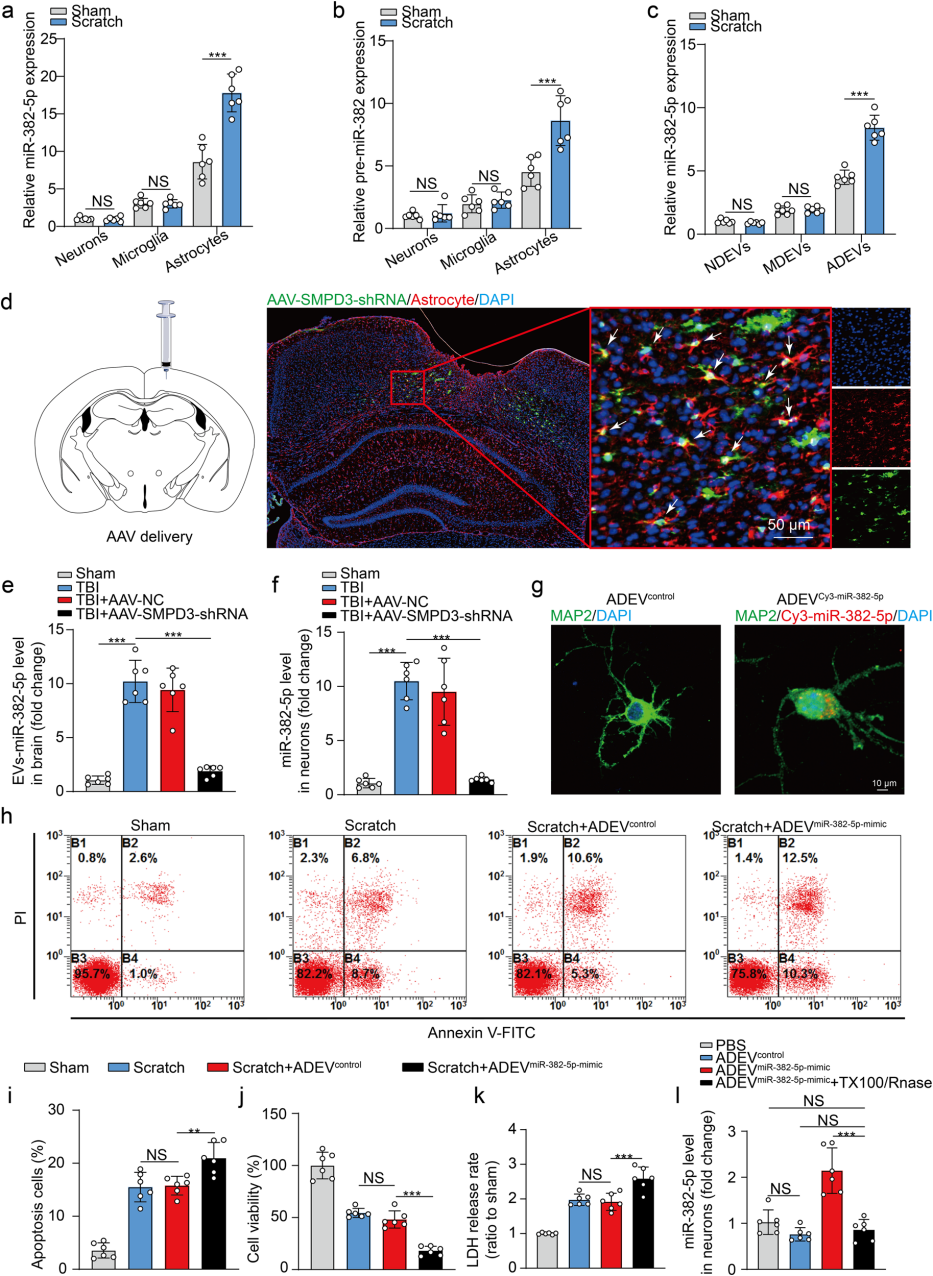
Internalization of astrocyte-derived EV-miRNA-382-5p promotes apoptosis in primary neurons. **a**, **b** Relative expression levels of miRNA-382-5p and premiR-382 in neurons, microglia and astrocytes at 24 h after scratch injury (*n* = 6 per group; Student’s *t* test). **c** Relative miRNA-382-5p expression levels among NDEVs, MDEVs, and ADEVs at 24 h after scratch injury (*n* = 6 per group; Student’s t test). **d** Representative images of immunofluorescence staining of mouse brain sections obtained 2 weeks after the stereotactic injection of AAV-SMPD3-shRNA containing a GFAP-specific promoter. Scale bars, 50 µm. **e** qRT‒PCR analysis of the level of EV-derived miRNA-382-5p in the perilesional cortex and hippocampus of TBI mice with or without astrocyte-specific knockdown of SMPD3 (*n* = 6 per group; one-way ANOVA). **f** qRT‒PCR analysis of the level of miRNA-382-5p in neurons isolated from TBI mice with or without astrocyte-specific knockdown of SMPD3 (*n* = 6 per group; one-way ANOVA). **g** Translocation of the extracellular vesicle Cy3-miR-382-5p from astrocytes to primary neurons. Confocal imaging of primary neurons. **h**–**k** ADEVs promoted apoptosis (**h**, **i**), decreased cell viability (**j**), and increased lactate dehydrogenase (LDH) (**k**) release from scratched primary neurons (*n* = 6 per group; one-way ANOVA). **l** Triton X-100/RNase treatment prevented the transfer of miRNA-382-5p from astrocytes into primary neurons (*n* = 6 per group; one-way ANOVA). The data are presented as the means ± SDs; *p < 0.05, **p < 0.01, ***p < 0.001, and NS not significant.

We cultured primary cortical microglia, astrocytes, and neurons and measured the expression of mature miRNA-382-5p and premiR-382 in these cells to investigate which cell type is responsible for the secretion of miRNA-382-5p-enriched EVs in the brain. As shown in Fig. 2a, b, astrocytes, but not microglia or neurons, presented significantly increased expression of both mature miRNA-382-5p and premiR-382 in a cell model of mechanical scratch injury compared with that in the sham group. These data were consistent with the qPCR results showing that miRNA-382-5p was highly enriched in astrocyte-derived EVs (ADEVs) and further increased in response to scratching stimuli but not in neuron-derived EVs (NDEVs) or microglia-derived EVs (MDEVs) (Fig. 2c).

The enzyme neutral sphingomyelinase 2 (nSMase2), which is derived from the SMPD3 gene, plays a crucial role in the synthesis and release of EVs^38,39^. We stereotaxically injected an adeno-associated virus (AAV)-SMPD3-shRNA containing a GFAP-specific promoter that specifically targets nSMase2 in astrocytes to further confirm that miRNA-382-5p is derived from astrocytes in TBI. The results of the immunofluorescence analysis revealed that 95.7% ± 2.6% (mean ± SE) of the SMPD3-shRNA^+^ cells expressed the astrocyte marker GFAP, indicating their astrocytic identity. In contrast, 66.23 ± 3.61% (mean ± SE) of GFAP^+^ cells could be traced by the SMPD3-shRNA (Fig. 2d), indicating high efficiency of AAV infection. We subsequently investigated the impact of AAV-SMPD3-shRNA infection on the concentration and diameter of EVs in mouse brain tissue and plasma using NTA. The results showed that AAV-SMPD3-shRNA significantly decreased the number of EVs in both the brain tissue (Supplementary Fig. 3a, b) and plasma of TBI mice (Supplementary Fig. 3d, e). However, no significant changes were observed in the size distribution of EVs following treatment with AAV-SMPD3-shRNA (Supplementary Fig. 3c,f). In marked contrast to the results from the mice injected with AAV-NC-shRNA, the inhibition of astrocytic EV secretion with AAV-SMPD3-shRNA significantly reduced the total EV-miR-382-5p levels in both whole-brain tissue (Fig. 2e) and isolated neurons (Fig. 2f) following TBI. Collectively, our in vivo and in vitro data provide evidence that the increase in EV-derived miR-382-5p expression originated from astrocytic EVs in the TBI model.

Moreover, the administration of AAV-SMPD3-shRNA effectively attenuated brain edema (Supplementary Fig. 4a) and improved neurological function (Supplementary Fig. 4b–d) following TBI. These findings suggest that astrocytes in the injured cortex are activated during the acute phase of TBI, giving rise to a neurotoxic subtype of cells that induce neuronal death by delivering injurious EVs to neurons. However, EVs encompass a diverse range of components, each performing distinct molecular functions. While AAV-SMPD3-shRNA inhibits the release of detrimental EV-derived miRNA-382-5p from astrocytes, it also impedes the secretion of other EV components, both harmful and beneficial. Therefore, we conducted subsequent experiments examining the effects of ADEV^miR-382-5p-mimic^ and AAV-miR-382-5pi to elucidate the mechanism underlying the neurotoxic effect specifically attributed to EV-miRNA-382-5p.

Astrocytes are critical for the trophic and metabolic support of neurons, mainly through astrocyte-secreted mediators, such as gliotransmitters, energy substrates and trophic factors^40,41^. We investigated whether ADEV-miRNA-382-5p may serve as a mediator of astrocyte–neuron crosstalk by transfecting astrocytes with Cy3-miRNA-382-5p-mimics (ADEV^Cy3-miR-382-5p^), a control plasmid (ADEV^control^), or PBS. Then, EVs were isolated from the astrocyte-conditioned medium and cocultured with primary neurons for 24 h. Strikingly, robust intracellular red fluorescence (Cy3) was observed in neurons cultured with conditioned medium from Cy3-miRNA-382-5p mimic-transfected astrocytes but not in neurons cultured with astrocytes transfected with ADEV^control^ (Fig. 2g). Moreover, ADEV^miR-382-5p-mimic^ significantly exacerbated neuronal apoptosis (Fig. 2h–i), reduced cell viability (Fig. 2j), and promoted lactate dehydrogenase (LDH) release (Fig. 2k).

We employed a vesicle–RNA degradation assay in which Triton X-100 and RNase were used as detergents to disrupt the membrane structure of EVs and digest RNA to further confirm that increased intracellular miRNA-382-5p resulted from the internalization of exogenous EV-miR-382-5p, the complete degradation of ADEV-miRNA-382-5p was observed (Fig. 2l). As expected, the increase in miRNA-382-5p expression in neurons treated with ADEV^miR-382-5p-mimic^ was completely abolished by vesicle-mediated RNA degradation. Taken together, the above data revealed that in response to brain injury, EV-miRNA-382-5p was derived mainly from astrocytes and could be internalized by neurons, indicating a potential role for EV-miRNA-382-5p in astrocyte–neuron crosstalk.

### Inhibition of miRNA-382-5p expression in astrocytes alleviates brain edema and neurological dysfunction after TBI

**Fig. 3:**
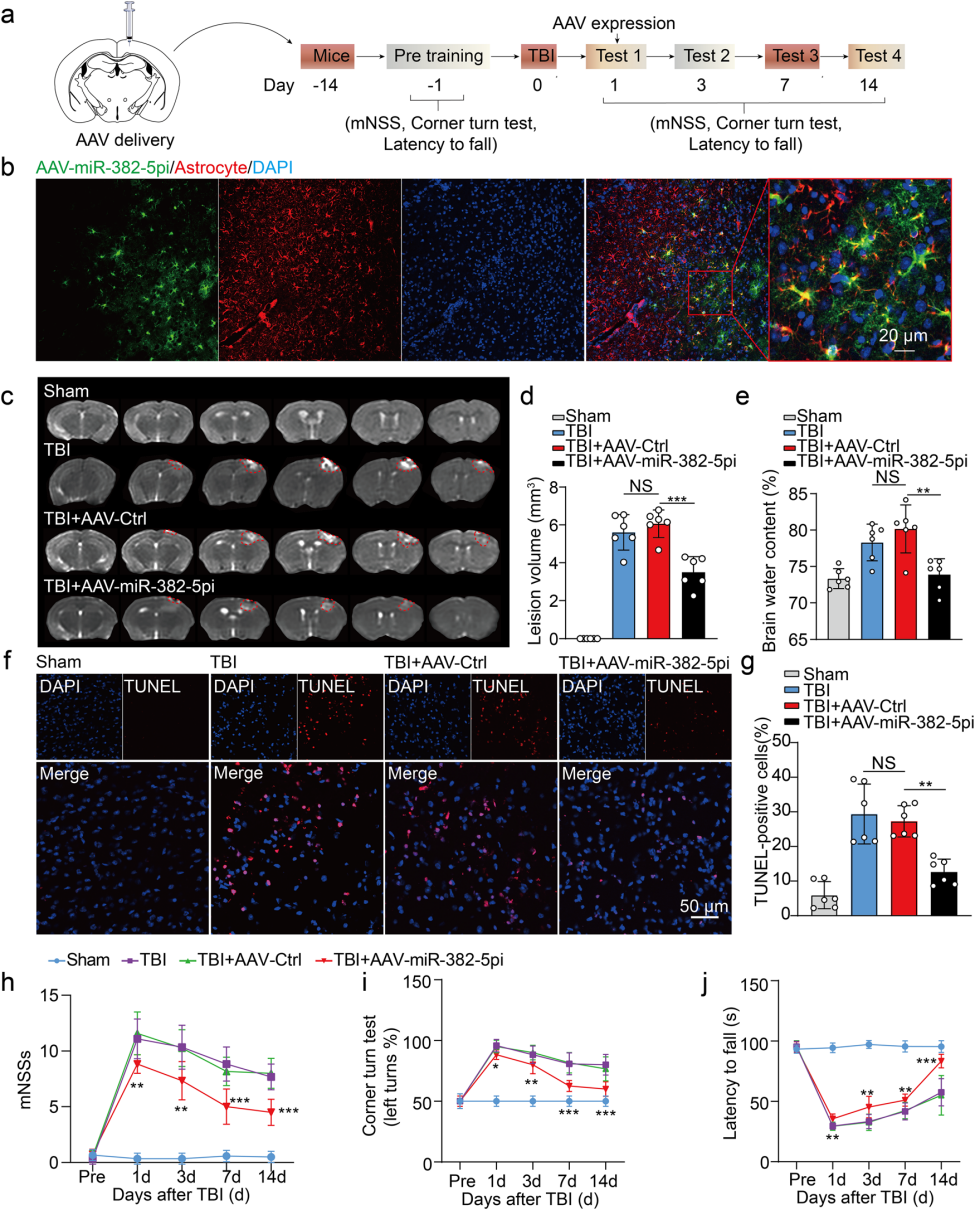
Astrocyte-specific inhibition of miRNA-382-5p can reduce brain tissue injury and the loss of neurological function after TBI. **a** Timeline for AAV injection, TBI induction, and behavioral assessments in the experimental study. **b** The image illustrates the localization of AAV-miR-382-5pi expression within the cortical region at 15 days postinjection. AAV-miR-382-5pi targets astrocytes. Viral GFP expression (green) colocalizes with an astrocytic marker (GFAP, red). Scale bar, 20 μm. **c**, **d** Representative MR images and statistical analysis of histological impairments (*n* = 6 per group; one-way ANOVA) at 24 h after TBI. **e** Statistical analysis of brain edema. **f**, **g** Representative images of TUNEL staining of the AAV-miR-382-5pi group or AAV-Ctrl group (*n* = 6 per group; one-way ANOVA). **h**–**j** Neurological function deficit scores in the AAV-miR-382-5pi group and the AAV-Ctrl group (*n* = 12 per group; two-way ANOVA). The data are presented as the means ± SDs; *p < 0.05, **p < 0.01, ***p < 0.001, and NS not significant.

We determined the functional role of miRNA-382-5p in TBI-associated secondary injury in mice by employing RNA interference-based miRNA-382-5p silencing in astrocytes using the AAV approach (AAV-miR-382-5pi). Damage to the right hemisphere (ipsilateral side to treatment) was induced 14 days after virus injection (Fig. 3a, b), and the degree of brain injury was measured by MRI 24 h after TBI (Fig. 3c). Interestingly, compared with the AAV-Ctrl-treated group, the AAV-miR-382-5pi-treated group presented significant reductions in the lesion volume (Fig. 3d), brain edema (Fig. 3e) and the proportion of TUNEL-positive cells (Fig. 3f, g). Consistent with these findings, in the functional recovery tests using modified neurological severity scores (mNSSs), the corner turn test, and the wire hanging test, also significant improvements were also observed in the TBI + AAV-miR-382-5pi-treated group compared with the TBI + AAV-Ctrl-treated group (Fig. 3h–j). Collectively, these results demonstrated that astrocyte-specific inhibition of miRNA-382-5p promoted the recovery of neurological function in the TBI model.

### Inhibition of miRNA-382-5p expression in astrocytes alleviates the neuronal mitochondrial imbalance after TBI

**Fig. 4:**
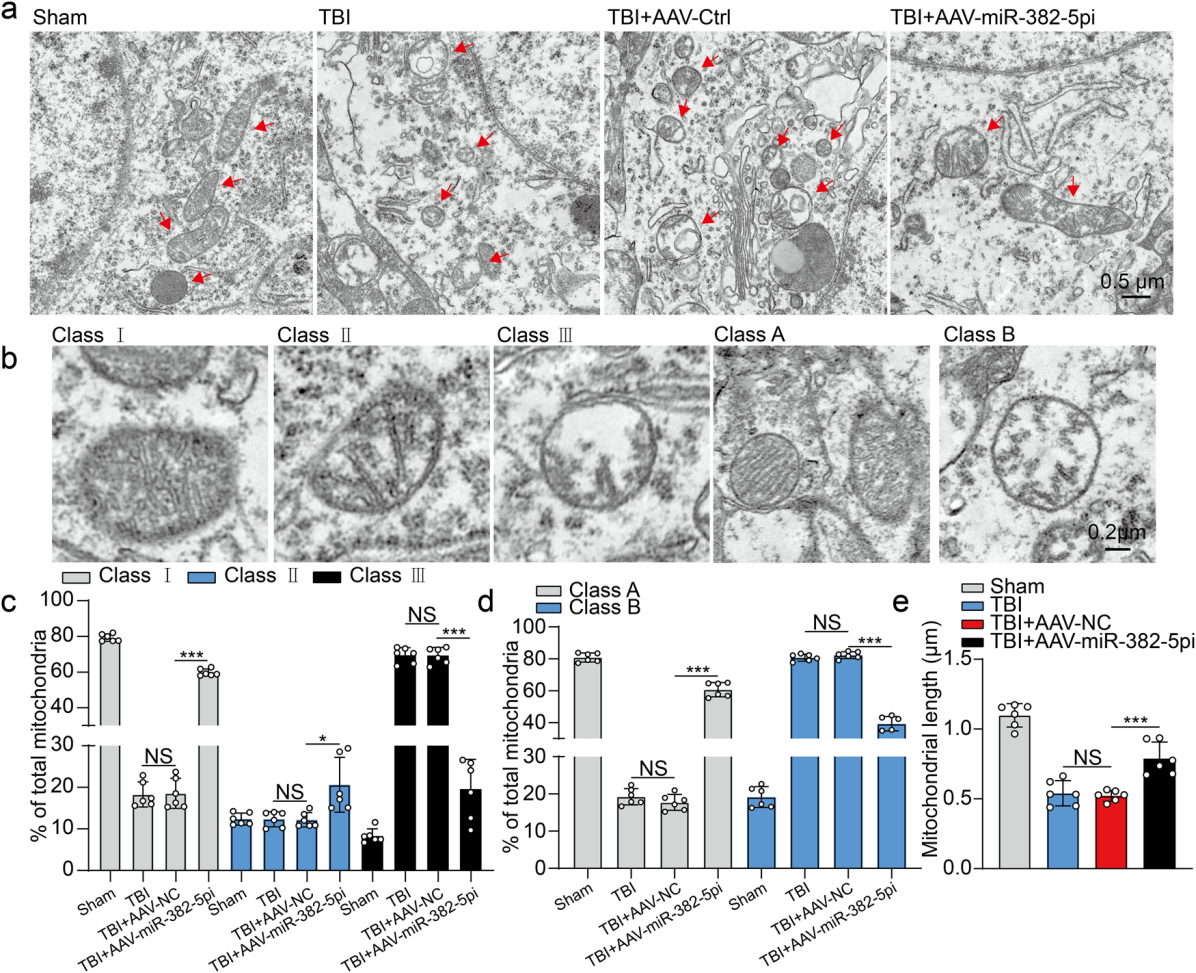
Astrocyte-specific inhibition of miRNA-382-5p reduces mitochondrial damage after TBI. **a** Representative electron microscopy (EM) images showing the number of mitochondrial cristae, the matrix density, and swelling in the perilesional cortex after TBI. **b** TEM images showing mitochondrial crista remodeling in the perilesional cortex of the AAV-miR-382-5pi group or AAV-Ctrl group. **c** Fifty randomly selected mitochondria per sample were scored according to the number of mitochondrial cristae and divided into three categories: more than four cristae (Class I), two or three cristae (Class II), and one or no cristae (Class III) per mitochondrion. The proportion of mitochondria in each class was calculated (*n* = 6 per group, one-way ANOVA). **d** Fifty randomly selected mitochondria per sample were scored according to matrix density and swelling and divided into two categories: dense matrix (Class A) and swollen mitochondria with a hypodense matrix (Class B). The proportion of mitochondria in each class was calculated (*n* = 6 per group, one-way ANOVA). **e** The length of the mitochondria in each group, which is represented by the mean length of 50 randomly selected mitochondria (*n* = 6 per group; one-way ANOVA). The data are presented as the means ± SDs; *p < 0.05, ***p < 0.001, and NS not significant.

Previous studies have shown that an imbalance in mitochondrial dynamics contributes to structural and functional damage in a mouse model of TBI^42,43^. We examined the ultrastructure of the mitochondria in the injured area via electron microscopy to determine whether elevated ADEV-miRNA-382-5p is involved in the mitochondrial imbalance. As shown in Fig. 4a, neuronal mitochondria in the sham group displayed considerable morphological uniformity, as represented by the comparable number of cristae in each mitochondrion. Notably, changes in mitochondrial morphology during apoptotic cell death, typified by the formation of short and round mitochondria, were observed in the TBI group (Fig. 4a), while treatment with AAV-miR-382-5pi significantly rescued the dynamics of mitochondria.

We accurately quantified the morphological changes in the mitochondria by categorizing the mitochondria into different types according to the number of cristae (Class I: more than four cristae; Class II: 2–3 cristae; Class III: no cristae or one crista) and the degree of mitochondrial swelling (Class A: a dense matrix; Class B: swollen mitochondria with a hypodense matrix)^44,45^ (Fig. 4b). The neurons in the injured cortex exhibited significant mitochondrial swelling, loss and destruction of the mitochondrial cristae, and reduced integrity of the mitochondrial membrane, with 18.29% of the mitochondria rated as Class I and 19.25% as Class A (Fig. 4c, d). Notably, treatment with AAV-miR-382-5pi significantly reduced the number of morphological changes in the mitochondria; 59.78% of the mitochondria were classified into Class I, and 60.68% were classified into Class A (Fig. 4c, d). Moreover, the reduction in the length of mitochondria in the TBI group was reversed in the AAV-miR-382-5pi-treated group (Fig. 4e). Taken together, these findings indicated that ADEV-miRNA-382-5p regulated mitochondrial morphology in response to TBI and that astrocyte-specific inhibition of miRNA-382-5p effectively counteracted the TBI-induced impairment of mitochondrial dynamics in neurons.

### The downregulation of OPA1 by ADEV-miRNA-382-5p promotes a neuronal mitochondrial imbalance

**Fig. 5:**
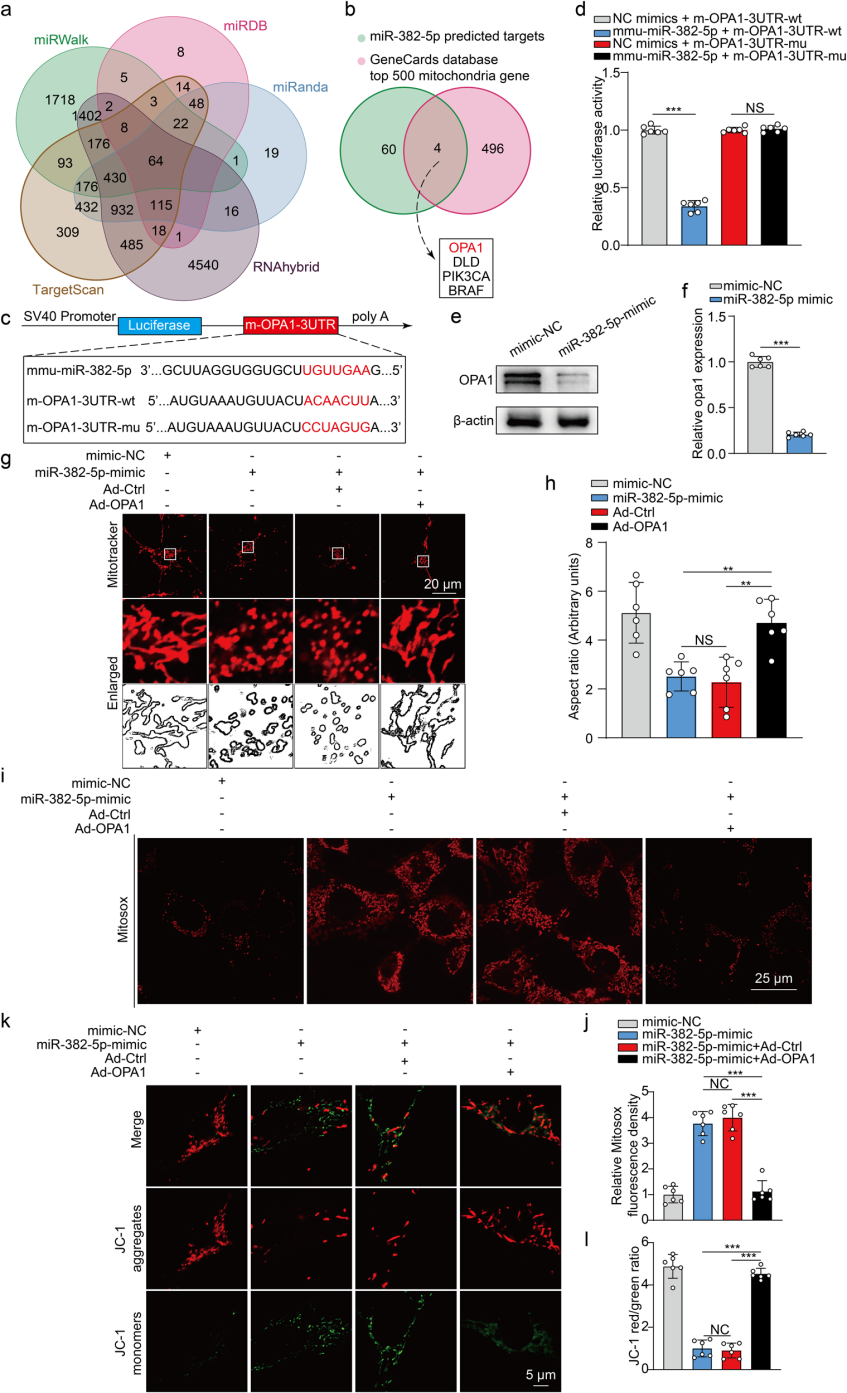
ADEV-miRNA-382-5p downregulates OPA1 expression in neuronal mitochondria in the context of a mitochondrial imbalance. **a** Overlap of potential targets of miRNA-382-5p predicted by the TargetScan, miRWalk, miRDB, RNAhybrid, and miRanda databases. **b** Venn diagram of putative target genes of miRNA-382-5p overlapping with mitochondria-related genes. **c** Wild-type and mutant OPA1 3′-UTR reporter constructs. **d** Luciferase reporter assay results for HEK293T cells cotransfected with the indicated wild-type or mutant 3’-UTR constructs and the miRNA-382-5p mimic (*n* = 6 per group; one-way ANOVA). **e**, **f** WB analysis and densitometric quantification of OPA1 levels in primary neurons transfected with or without the miRNA-382-5p mimic (*n* = 6 per group; Student’s *t* test). **g** MitoTracker was used to label mitochondria in primary neurons, mitochondrial morphology was examined via fluorescence microscopy, and mitochondrial morphological features were quantified (aspect ratios) using ImageJ software. **h** The boxed area next to each micrograph shows an magnified version of the white square (*n* = 6 per group, one-way ANOVA). **i**, **j** Representative images of MitoSOX fluorescence and quantitative comparison of mitochondria-derived ROS levels (*n* = 6 per group; one-way ANOVA). **k**, **l** Representative images of fluorescence staining and quantitative comparison of JC-1 aggregates (red fluorescence) and JC-1 monomers (green fluorescence) in cultured primary neurons from different experimental groups (*n* = 6 per group; one-way ANOVA). The data are presented as the means ± SDs; **p < 0.01, ***p < 0.001, and NS not significant.

Previous studies have shown that miRNA-382-5p can target intracellular phosphatase and tensin homolog (PTEN) and nuclear receptor subfamily 3 group C member 1^46^, but the molecular mechanism underlying the miRNA-382-5p-mediated regulation of mitochondrial dynamics remains unknown. We addressed this question by analyzing five databases (TargetScan, miRanda, RNAhybrid, miRDB, and miRWalk)^47–49^ and found that 64 genes could be potential targets (Fig. 5a). Furthermore, by intersecting with mitochondrial genes from the GeneCards database^50^ (Fig. 5b), OPA1, DLD, PIK3CA and BRAF were the most likely candidates. Notably, OPA1, a member of the “mitochondria-shaping” protein family, plays a crucial role in maintaining mitochondrial stability and preventing apoptosis by participating in mitochondrial crista remodeling, maintaining mtDNA integrity, regulating cytochrome c release, and modulating oxidative phosphorylation^44^. Additionally, our data revealed a significant downregulation of OPA1 expression in the acute phase following TBI (Supplementary Fig. 5a, b), which is consistent with previous reports^51^.

We employed a luciferase reporter assay to determine whether and how miRNA-382-5p binds the 3’-untranslated region (3’ UTR) of OPA1. The wild-type OPA1 3’ UTR (WT) and mutant OPA1 3’ UTR (MUT), which may contain the miRNA-382-5p binding site, were inserted into the luciferase reporter vector pSI-Check-2 and cotransfected into HEK293T cells with miRNA-382-5p or miR-NC (Fig. 5c). Interestingly, the activity of luciferin was significantly inhibited by the overexpression of miRNA-382-5p (Fig. 5d), resulting in a notable reduction in OPA1 protein expression in primary neurons (Fig. 5e, f).

We treated primary neurons with a GV314 adenoviral vector expressing OPA1 (Ad-OPA1) to determine the potential role of OPA1 expression in mitochondrial dynamics. Strikingly, a reduced aspect ratio for MitoTracker staining revealed increased fragmentation of mitochondria in the miRNA-382-5p mimic-treated group, whereas reintroducing OPA1 with Ad-OPA1 attenuated miRNA-382-5p-induced mitochondrial fragmentation (Fig. 5g, h). Moreover, OPA1 overexpression significantly reduced ROS production (Fig. 5i, j) and mitochondrial membrane depolarization (Fig. 5k, l) compared with those in the miRNA-382-5p-treated groups, indicating that increased levels of OPA1 may restore mitochondrial function.

**Fig. 6:**
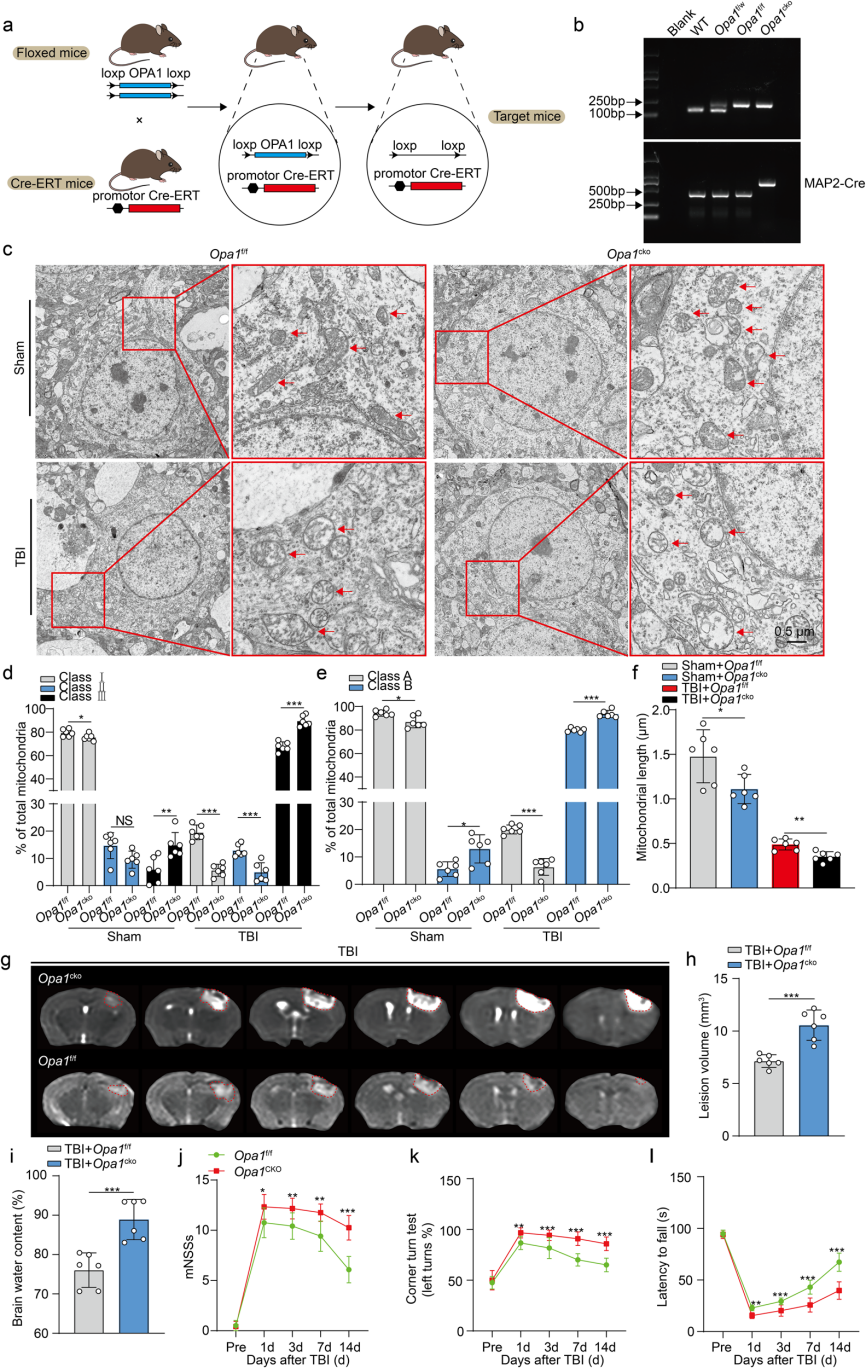
Neuronal-specific knockout of OPA1 aggravated mitochondrial damage and neurological dysfunction after TBI. **a** Cartoon illustrating the process used to construct OPA1 conditional knockout mice. **b** Results showing the identification of the neuron-specific OPA1 conditional knockout mice. **c** TEM images showing cortical mitochondrial crista remodeling in the *Opa1*^f/f^ group or *Opa1*^CKO^ group subjected to sham surgery or TBI. **d** Fifty randomly selected mitochondria per sample were scored in three categories (*n* = 6 per group, one-way ANOVA): more than four cristae (Class I), between two and three cristae (Class II), and one or no cristae (Class III) per mitochondrion. **e** Fifty mitochondria per sample were scored according to matrix density and swelling (*n* = 6 per group, one-way ANOVA): dense matrix (Class A) and swollen mitochondria with a hypodense matrix (Class B). **f** The length of the mitochondria in each group, which is represented by the mean length of 50 randomly selected mitochondria (*n* = 6 per group; one-way ANOVA). **g**, **h** Representative MR images and statistical analysis of histological impairments at 24 h after TBI (*n* = 6 per group; Student’s *t* test). **i** Statistical analysis of brain edema. *n* = 6 per group; Student’ s *t* test) **j**–**l** The neurological function deficit scores of the *Opa1*^f/f^ group or *Opa1*^CKO^ group (*n* = 12 per group, two-way ANOVA). The data are presented as the means ± SDs; *p < 0.05, **p < 0.01, and ***p < 0.001.

We generated neuron-specific *Opa1* knockout (*Opa1*^CKO^) mice by crossing *Opa1*^flox/flox^ (*Opa1*^f/f^) mice with *Map2-creERT2* mice to further elucidate the relationship between OPA1 and miRNA-382-5p and investigate the role of OPA1 in mitochondria (Fig. 6a, b). We subsequently assessed whether the neuroprotective effect of AAV-miRNA-382-5pi on neuronal damage following TBI was abolished in *Opa1*^CKO^ mice. Our results indicated that AAV-miRNA-382-5pi treatment failed to alleviate functional deficits in *Opa1*^CKO^ mice following TBI, as evaluated by the mNSS, corner turn test, and wire suspension test (Supplementary Fig. 6a–c), suggesting direct targeting of OPA1 by miRNA-382-5p. Taken together, these findings indicated that miRNA-382-5p directly targeted OPA1 and that changes in OPA1 expression may be responsible for the miRNA-382-5p-induced disturbance of mitochondrial morphology and function in neurons.

### Conditional knockout of OPA1 in neurons aggravates mitochondrial damage, apoptosis and neurological deficits after TBI

The impact of OPA1 on alterations in mitochondrial function following TBI was subsequently investigated. As expected, although mitochondrial damage was observed in both the *Opa1*^f/f^ and *Opa1*^CKO^ mice 24 h after TBI (Fig. 6c), the *Opa1*^CKO^ mice presented more severe mitochondrial damage, as evidenced by the more severe loss of the mitochondrial cristae and mitochondrial membrane integrity and increased mitochondrial fragmentation (Fig. 6d, e), accompanied by a markedly shortened mitochondrial length (Fig. 6f). In addition to these morphological changes, we detected increased ROS levels (Supplementary Fig. 7a, b), increased malondialdehyde (MDA) levels (Supplementary Fig. 7c) and decreased manganese superoxide dismutase (MnSOD) activity (Supplementary Fig. 7d) in the cortex of *Opa1*^CKO^ mice compared with their littermate controls. As a result of morphological and functional abnormalities in mitochondria, *Opa1*^CKO^ mice presented a significant increase in the proportion of apoptotic cells (Supplementary Fig. 8a, b), an increased lesion volume (Fig. 6g, h) and an increased severity of cerebral edema (Fig. 6i). Consistent with these data, the functional deficits evaluated using the mNSS, the corner turn test, and the wire suspension test revealed an increased severity of neurological impairment in *Opa1*^CKO^ mice (Fig. 6j–l). Taken together, these findings suggested that OPA1 contributed to the maintenance of mitochondrial dynamics, which was pivotal for the optimal function of mitochondria and cell fate.

### Restoring mitochondrial OPA1 expression with engineered EVs alleviates TBI-induced pathogenesis

**Fig. 7:**
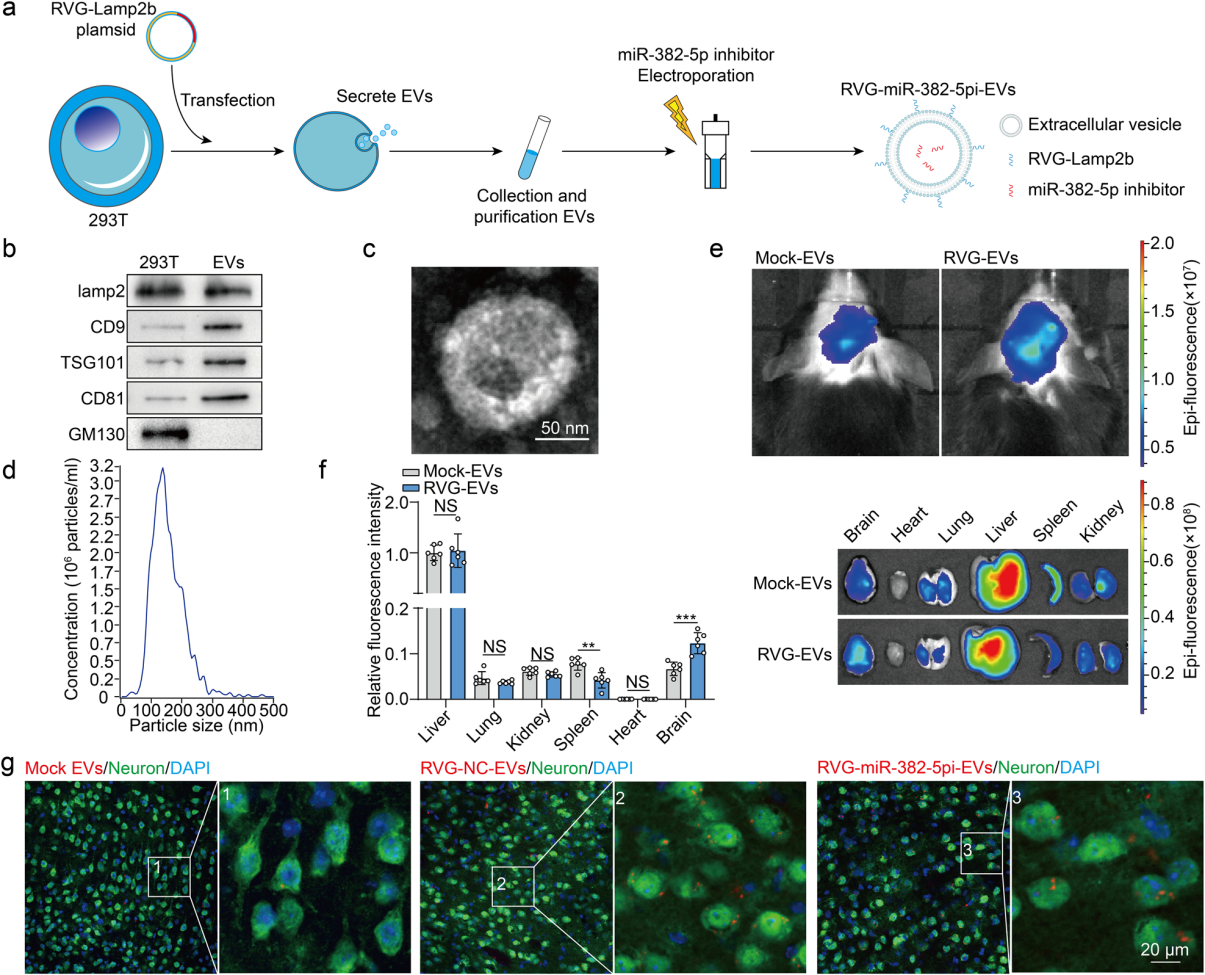
Characterization of engineered RVG-EVs. **a** Schematic representation of the production and harvest of EVs for targeted miR-382-5pi delivery. **b** WB analysis of lamp2, TSG101, CD9, CD81, and GM130 levels in HEK293T cell-derived EVs. **c** Representative electron micrograph of isolated extracellular vesicles. **d** Histograms displaying the size distribution and number of particles per milliliter for EVs extracted from HEK293T cell cultures. **e** Distribution of DiR-labeled mock EVs or RVG-EVs in mice and different organs at 24 h after injection, as determined via live fluorescence imaging. **f** The boxed graph shows the locations of the six analyzed organs (*n* = 6 per group; Student’s *t* test). **g** Confocal microscopy images of PKH26-labeled RVG-EVs or PKH26-labeled mock-treated EVs. Red fluorescent spots indicate PKH26-labeled EVs, and green fluorescence represents neurons (NeuN^+^). The data are presented as the means ± SDs; *p < 0.05, **p < 0.01, ***p < 0.001, and NS not significant.

Engineered modified EVs could serve as efficient drug delivery carriers because of their low immunogenicity, limited biodegradability, minimal toxicity, and ability to cross the blood‒brain barrier^52^. Moreover, surface modification of EVs with specific peptides can allow them to selectively target and deliver cargo to specific tissues and organs^53,54^. Thus, we developed neuron-targeting rabies virus glycoprotein (RVG)-EVs by binding a specific RVG peptide to the membrane protein Lamp2b on EVs derived from HEK293T cells^55^ (Fig. 7a). These RVG-EVs were collected from the supernatant of cultured cells via gradient centrifugation and verified by nanoparticle analysis, electron microscopy, and WB (Fig. 7b–d).

RVG-EVs and mock-EVs were labeled with 200 μg of DiR and injected into TBI mice via the tail vein to verify the targeting efficiency of this drug delivery system. The intensity and distribution of fluorescence were subsequently assessed using the Xenogen IVIS imaging system at 24 h postinjection (Fig. 7e). The quantitative analysis revealed greater accumulation of EVs in the liver than in other organs (brain, heart, lungs, liver, spleen, or kidneys). Notably, more EVs accumulated in the brain following RVG-EVs treatment than following mock-EVs treatment (Fig. 7f), suggesting the intrinsic affinity of RVG-EVs for the injured brain. Next, miRNA-382-5pi or negative control (NC) (miRNA-382-5pi-NC) was encapsulated in RVG-EVs via electroporation and injected into TBI mice through the tail vein. The red signal of PKH26-labeled RVG-EVs was localized in neurons (Neun+) at 24 h postinjection (Fig. 7g), confirming the neuron-targeting ability of RVG-EVs.

**Fig. 8:**
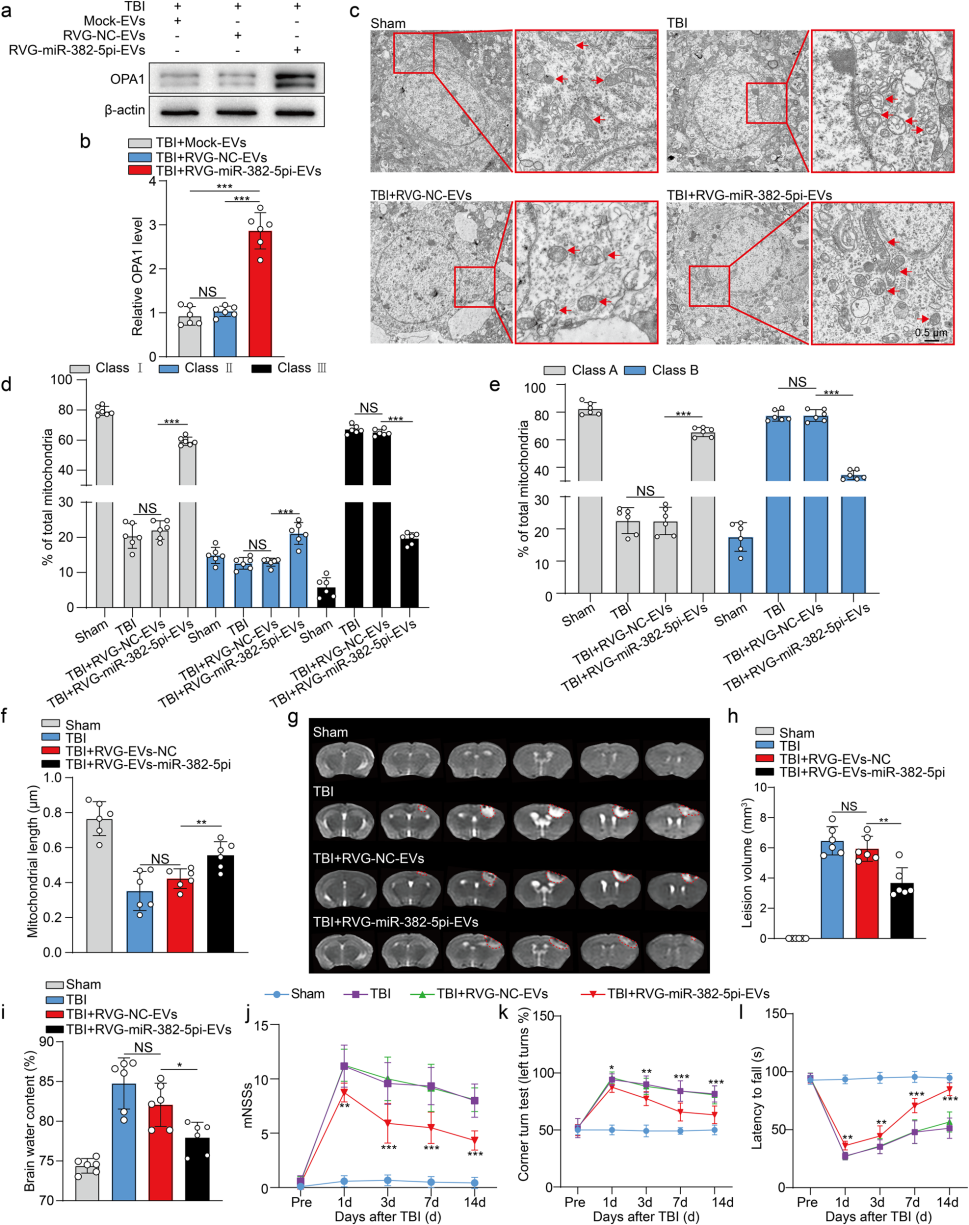
RVG-miR-382-5pi-EVs alleviate mitochondrial damage and improve neurological function after TBI. **a**, **b** WB analysis and densitometric quantification of the levels of the OPA1 protein in the perilesional cortex of mice injected with or without RVG-miR-382-5pi-EVs (*n* = 6 per group; one-way ANOVA). **c** TEM images showing that the loss of mitochondrial cristae in the perilesional cortex was inhibited in the RVG-NC-EVs group and the RVG-miR-382-5pi-EVs group. **d** Fifty mitochondria per sample were assigned to three categories: more than four cristae (Class I), between two and three cristae (Class II), and one or no cristae (Class III) per mitochondrion (*n* = 6 per group, one-way ANOVA). **e** Fifty mitochondria per sample were scored according to matrix density and swelling: dense matrix (Class A) and swollen mitochondria with a hypodense matrix (Class B) (*n* = 6 per group, one-way ANOVA). **f** Representative quantitative results of the length of 50 mitochondria per experiment (*n* = 6 per group; one-way ANOVA). **g**, **h** Representative MR images and statistical analysis of histological impairments at 24 h after TBI (*n* = 6 per group; one-way ANOVA). **i** Statistical analysis of brain edema (*n* = 6 per group, one-way ANOVA). **j‒l** Neurological function deficit scores of the RVG-NC-EVs group and RVG-miR-382-5pi-EVs group (*n* = 12 per group, two-way ANOVA). The data are presented as the means ± SDs; *p < 0.05, **p < 0.01, ***p < 0.001, and NS not significant.

As expected, RVG-miR-382-5pi-EVs-treated mice presented significantly higher OPA1 expression in the cerebral cortex than mock-EVs- and RVG-NC-EVs-treated mice (Fig. 8a, b). Consistently, changes in mitochondrial morphology, including the percentages of Class I and Class A mitochondria and the length of mitochondria, were markedly reversed by RVG-miR-382-5pi-EVs treatment (Fig. 8c–f), suggesting that RVG-miR-382-5pi-EVs alleviated TBI-induced damage to the mitochondrial morphology by restoring OPA1 expression.

We evaluated the lesion volume, severity of brain edema and degree of cellular apoptosis in mice to further investigate the neuroprotective effects of RVG-miR-382-5pi-EVs. Compared with the RVG-NC-EVs group, the group treated with RVG-miR-382-5pi-EVs presented a significantly reduced lesion volume (Fig. 8g, h), reduced brain water content (Fig. 8i), and decreased apoptosis (Supplementary Fig. 9a, b).

The impact of RVG-miR-382-5pi-EVs on functional recovery in TBI model mice was subsequently assessed. Intravenous injections of RVG-miR-382-5pi-EVs or RVG-NC-EVs every other day for 12 consecutive days significantly ameliorated neurological impairments following TBI (Fig. 8j–l). Overall, these in vivo findings suggested that RVG-miRNA-382-5pi-EV therapy may protect against neuronal damage and promote neurological functional recovery after TBI.

### miRNA-382-5p in circulating EVs may be a diagnostic marker for predicting the outcomes of TBI in patients

Having established the critical role of EV-derived miRNA-382-5p in a mouse model of TBI, we wondered whether EV-derived miRNA-382-5p could be used as a prognostic marker in patients. The levels of miRNA-382-5p in the circulating EVs of TBI patients were evaluated via real-time quantitative PCR (qPCR) to address this question. Strikingly, the data revealed significantly higher miRNA-382-5p levels in the circulating EVs of patients with a poor prognosis (6-month Glasgow Outcome Scale (GOS) score≤3) than in those with a good prognosis (Supplementary Fig. 10a and Supplementary Table 2). A receiver operating characteristic (ROC) analysis was subsequently conducted to evaluate the diagnostic efficacy of miRNA-382-5p. The area under the ROC curve (AUC) was determined to be 0.754, with a sensitivity of 90% and a specificity of 63.33% (Supplementary Fig. 10b). Upon conducting an additional assessment through multivariate logistic regression analysis, miRNA-382-5p remained a significant determinant of the neurological prognosis of patients with TBI at six months following the onset of the condition, as measured by GOS scores (Supplementary Table 3). These findings collectively indicate that miRNA-382-5p has significant value in predicting the outcomes of TBI.

## Discussion

Here, we show that astrocyte-derived miRNA-382-5p-enriched EVs play a pivotal role in TBI-related neuronal damage by suppressing the expression of OPA1, resulting in an imbalance in mitochondrial dynamics, neuronal apoptosis, and neurological deficits. Additionally, we developed an engineered EV intervention system that exerts remarkable therapeutic effects and shows potential for clinical translation.

Despite significant advancements in medical care over the past few decades^1,2^, misdiagnosis often occurs in patients with mild TBI^56^, but accurately assessing patients with moderate to severe TBI during the hyperacute and acute stages also presents significant challenges^57^. Therefore, improving the clinical diagnosis and prognosis of TBI by identifying relevant variables or biomarkers that can facilitate clinical interventions and identify individuals at increased risk for poor recovery and chronic sequelae is imperative. In the present study, we hypothesized that EV-miRNA-382-5p may serve as a potential biomarker in TBI patients. In support of these findings, a systematic analysis of the plasma EV-miRNA profiles of TBI patients and tissue EV-miRNA profiles of a mouse model of TBI revealed that the concentrations of circulating EVs increased significantly after TBI, which is consistent with previous reports^58,59^, and we identified miRNA-382-5p as a potential plasma biomarker for the early diagnosis of TBI (Supplementary Fig. 10b).

EVs derived from astrocytes have the potential to exert either detrimental or advantageous effects on various types of cells after brain injury^60^. For example, the protein and miRNA cargo in astrocyte-derived EVs promote the transmigration of leukocytes into the brain by inhibiting peroxisome proliferator-activated receptor α (PPARα) in response to inflammatory brain injury^59^. Astrocyte-derived EV-miRNA-873a-5p is involved in the microglial M2 phenotypic transformation by inhibiting the phosphorylation of ERK and NF-κB p65, thereby exerting an antineuroinflammatory effect on TBI^61^. Our previous study revealed that astrocyte-derived EVs-miRNA-143-3p can be shuttled into brain microvascular endothelial cells (BMECs) after intracerebral hemorrhage and facilitate the transendothelial migration of circulating neutrophils into the brain by inducing the expression of cell adhesion molecules (CAMs) in targeted BMECs^62^. Moreover, the current study identified astrocyte-derived EV-derived miRNA-382-5p as a key mediator of neuronal apoptosis by regulating mitochondrial homeostasis, supporting the pivotal role of astrocyte-derived EVs in traumatic brain injury.

Although previous studies have shown that miRNA-382-5p is involved mainly in cancer development, its precise role remains controversial. For example, miRNA-382-5p promotes cell invasion in hepatocellular carcinoma by targeting phosphatase and tensin homolog (PTEN)^63^, whereas other researchers have shown that miRNA-382-5p inhibits the development of osteosarcoma by negatively regulating VEZF1^64^. Interestingly, the level of miRNA-382-5p was increased in myocardial tissues in a mouse model of acute myocardial infarction, suggesting a potential role for miRNA-382-5p in the modulation of cell apoptosis^65^. Consistent with this study, our data revealed that increased levels of miRNA-382-5p in neurons resulted in excessive neuronal damage in a mouse model of TBI. Mechanistically, miRNA-382-5p participated in regulating mitochondrial homeostasis by targeting OPA1, a mitochondrial dynamin-like GTPase anchored to the inner mitochondrial membrane that is essential for maintaining normal mitochondrial morphology^66^. Considering that mitochondria are an important intracellular source of ROS generation^67,68^, the interaction between miRNA-382-5p and the OPA1 mRNA may be involved in the excessive generation of ROS in neurons after TBI.

Although astrocyte-specific inhibition of miRNA-382-5p by AAV-miR-382-5pi contributed to mitigating secondary injury after TBI, these findings provide evidence that miRNA-382-5p may serve as a promising therapeutic target for TBI. The clinical translation of gene editing tools such as AAVs and lentiviruses is limited by safety concerns and the delayed onset of their effects^69^. Additionally, the effectiveness of interventions utilizing crude small interfering RNAs is compromised by their instability in the body due to susceptibility to multiple ribonucleases and the reticuloendothelial system^70,71^. However, EVs have the attributes of low immunogenicity, low biodegradability, and low toxicity while also possessing the ability to traverse the BBB, making them efficient drug delivery carriers^52,53^. Notably, the use of EVs to deliver miRNAs may protect them from RNase-mediated degradation and mitigate interference from peripheral factors^52^. For example, EV-mediated delivery of miR-17-92 has been shown to enhance neuroplasticity and improve functional recovery following stroke^23^. Consistent with these findings, we successfully delivered neuron-targeting miRNA-382-5p inhibitors through RVG-miR-382-5pi-EVs, which effectively improved mitochondrial function, attenuated oxidative stress, reduced neuronal apoptosis, and alleviated neurological deficits following TBI. These findings underscore the potential clinical applications of engineered EVs for the efficient delivery of miRNA-382-5pi for TBI treatment.

However, several unresolved issues remain. Due to the limited sample size, we were unable to conduct a comprehensive subgroup analysis investigating the heterogeneity of astrocyte-derived EVs in patients with varying degrees of disease severity and different injury factors. The diverse pathological mechanisms observed in different TBI patients make identifying a single biomarker with consistent diagnostic and prognostic value for all TBI patients challenging^72^. Therefore, future research may focus on exploring the combination of multiple diagnostic markers tailored to specific conditions in TBI patients. The primary objective of this study was to investigate the characteristics of astrocyte-derived EVs in individuals with identified brain tissue injuries and explore the translational potential of targeting these EVs to prevent neuronal death. However, the role of astrocyte-secreted EVs in concussion patients remains incompletely understood. Considering that closed and mild head injuries such as concussions are more prevalent worldwide than penetrating craniocerebral injuries are, determining whether EV-miRNAs have diagnostic or therapeutic value is crucial, specifically for concussion patients.

In summary, we showed that astrocyte‒neuron crosstalk through the EV-miR-382-5p‒OPA1 signaling pathway plays a critical role in TBI-associated neuronal apoptosis. Importantly, we detected a significant increase in plasma EV-miR-382-5p levels in not only a mouse model of TBI but also human patients, while the inhibition of miRNA-382-5p through RVG-EV-mediated delivery effectively protected neurons from TBI. Taken together, our findings indicate a role for EV-miRNA-382-5p in clinical diagnosis, suggesting that EV-miRNA-382-5p has therapeutic potential for treating TBI.

## Supplementary information

Supplementary Information

## Data Availability

The data used to support the findings of this study are available from the corresponding author upon request.

## References

[1] Kong, L. Z., Zhang, R. L., Hu, S. H. & Lai, J. B. Military traumatic brain injury: a challenge straddling neurology and psychiatry. *Mil. Med. Res.* **9**, 2 (2022). 34991734 10.1186/s40779-021-00363-y PMC8740337

[2] Maas et al. Traumatic brain injury: progress and challenges in prevention, clinical care, and research. *Lancet Neurol.* **21**, 1004 (2022). 36183712 10.1016/S1474-4422(22)00309-X PMC10427240

[3] Huang, L. et al. Maintaining Drosha expression with Cdk5 inhibitors as a potential therapeutic strategy for early intervention after TBI. *Exp. Mol. Med.* **56**, 210 (2024). 38200156 10.1038/s12276-023-01152-4 PMC10834983

[4] Capizzi, A., Woo, J. & Verduzco-Gutierrez, M. Traumatic brain injury: an overview of epidemiology, pathophysiology, and medical management. *Med. Clin. North Am.* **104**, 213 (2020). 32035565 10.1016/j.mcna.2019.11.001

[5] Zhao, J. et al. Mitochondria transplantation protects traumatic brain injury via promoting neuronal survival and astrocytic BDNF. *Transl. Res.* **235**, 102 (2021). 33798765 10.1016/j.trsl.2021.03.017

[6] Fesharaki-Zadeh, A. Oxidative stress in traumatic brain injury. *Int. J. Mol. Sci.* **23**, 13000 (2022). 36361792 10.3390/ijms232113000 PMC9657447

[7] Kilbaugh, T. J. et al. Mitochondrial bioenergetic alterations after focal traumatic brain injury in the immature brain. *Exp. Neurol.* **271**, 136 (2015). 26028309 10.1016/j.expneurol.2015.05.009 PMC4586357

[8] Simon, D. W. et al. The far-reaching scope of neuroinflammation after traumatic brain injury. *Nat. Rev. Neurol.* **13**, 171 (2017). 28186177 10.1038/nrneurol.2017.13 PMC5675525

[9] Allen, N. J. & Eroglu, C. Cell biology of astrocyte-synapse interactions. *Neuron* **96**, 697 (2017). 29096081 10.1016/j.neuron.2017.09.056 PMC5687890

[10] Alhadidi, Q. M. et al. Astrocytes in functional recovery following central nervous system injuries. *J. Physiol*. **602**, 3069 (2023). 10.1113/JP284197 PMC11421637 37702572

[11] Burda, J. E., Bernstein, A. M. & Sofroniew, M. V. Astrocyte roles in traumatic brain injury. *Exp. Neurol.* **275**, 305 (2016). 25828533 10.1016/j.expneurol.2015.03.020 PMC4586307

[12] Hayakawa, K. et al. Transfer of mitochondria from astrocytes to neurons after stroke. *Nature* **535**, 551 (2016). 27466127 10.1038/nature18928 PMC4968589

[13] Guttenplan, K. A. et al. Neurotoxic reactive astrocytes induce cell death via saturated lipids. *Nature* **599**, 102 (2021). 34616039 10.1038/s41586-021-03960-y PMC12054010

[14] Yuan, M. & Wu, H. Astrocytes in the traumatic brain injury: the good and the bad. *Exp. Neurol.* **348**, 113943 (2022). 34863998 10.1016/j.expneurol.2021.113943

[15] Hu, Q. et al. Extracellular vesicles in the pathogenesis and treatment of acute lung injury. *Mil. Med. Res* **9**, 61 (2022). 36316787 10.1186/s40779-022-00417-9 PMC9623953

[16] Rigg, E. et al. Inhibition of extracellular vesicle-derived miR-146a-5p decreases progression of melanoma brain metastasis via Notch pathway dysregulation in astrocytes. *J. Extracell. Vesicles* **12**, e12363 (2023). 37759347 10.1002/jev2.12363 PMC10533779

[17] Zhang, L. et al. Targeted elimination of senescent cells by engineered extracellular vesicles attenuates atherosclerosis in ApoE(−/−) mice with minimal side effects. *Theranostics* **13**, 5114 (2023). 37771781 10.7150/thno.87484 PMC10526664

[18] Takahashi, Y. & Takakura, Y. Extracellular vesicle-based therapeutics: extracellular vesicles as therapeutic targets and agents. *Pharm. Ther.* **242**, 108352 (2023). 10.1016/j.pharmthera.2023.108352 36702209

[19] Lin, Z. et al. Circulating MiRNA-21-enriched extracellular vesicles promote bone remodeling in traumatic brain injury patients. *Exp. Mol. Med.* **55**, 587 (2023). 36869070 10.1038/s12276-023-00956-8 PMC10073188

[20] Zhao, H. et al. Small extracellular vesicles from brown adipose tissue mediate exercise cardioprotection. *Circ. Res.* **130**, 1490 (2022). 35387487 10.1161/CIRCRESAHA.121.320458

[21] Kim, G. et al. Systemic delivery of microRNA-21 antisense oligonucleotides to the brain using T7-peptide decorated exosomes. *J. Control Release* **317**, 273 (2020). 31730913 10.1016/j.jconrel.2019.11.009

[22] Ge, X. et al. Increased microglial exosomal miR-124-3p alleviates neurodegeneration and improves cognitive outcome after rmTBI. *Mol. Ther.* **28**, 503 (2020). 31843449 10.1016/j.ymthe.2019.11.017 PMC7001001

[23] Xin, H. et al. MicroRNA cluster miR-17-92 cluster in exosomes enhance neuroplasticity and functional recovery after stroke in rats. *Stroke* **48**, 747 (2017). 28232590 10.1161/STROKEAHA.116.015204 PMC5330787

[24] Hou, Z. et al. Longterm exercise-derived exosomal miR-342-5p: a novel exerkine for cardioprotection. *Circ. Res* **124**, 1386 (2019). 30879399 10.1161/CIRCRESAHA.118.314635

[25] Guyon, N. et al. Anti-PD1 therapy induces lymphocyte-derived exosomal miRNA-4315 release inhibiting Bim-mediated apoptosis of tumor cells. *Cell Death Dis.* **11**, 1048 (2020). 33311449 10.1038/s41419-020-03224-z PMC7733505

[26] Xiong, Y., Mahmood, A. & Chopp, M. Animal models of traumatic brain injury. *Nat. Rev. Neurosci.* **14**, 128 (2013). 23329160 10.1038/nrn3407 PMC3951995

[27] Rubin, T. G. & Lipton, M. L. Sex differences in animal models of traumatic brain injury. *J. Exp. Neurosci.* **13**, 1179069519844020 (2019). 31205421 10.1177/1179069519844020 PMC6537488

[28] Hosen, M. R. et al. Circulating microRNA-122-5p is associated with a lack of improvement in left ventricular function after transcatheter aortic valve replacement and regulates viability of cardiomyocytes through extracellular vesicles. *Circulation* **146**, 1836 (2022). 35862223 10.1161/CIRCULATIONAHA.122.060258

[29] Liu, S. et al. M1-like macrophage-derived exosomes suppress angiogenesis and exacerbate cardiac dysfunction in a myocardial infarction microenvironment. *Basic Res. Cardiol.* **115**, 22 (2020). 32112145 10.1007/s00395-020-0781-7

[30] Vella, L. J. et al. A rigorous method to enrich for exosomes from brain tissue. *J. Extracell. Vesicles* **6**, 1348885 (2017). 28804598 10.1080/20013078.2017.1348885 PMC5533148

[31] Fan, C. et al. Microglia secrete miR-146a-5p-containing exosomes to regulate neurogenesis in depression. *Mol. Ther.* **30**, 1300 (2022). 34768001 10.1016/j.ymthe.2021.11.006 PMC8899528

[32] Zhang, S. et al. Adiponectin/AdiopR1 signaling prevents mitochondrial dysfunction and oxidative injury after traumatic brain injury in a SIRT3 dependent manner. *Redox Biol.* **54**, 102390 (2022). 35793583 10.1016/j.redox.2022.102390 PMC9287731

[33] Del Dotto, V. et al. OPA1 isoforms in the hierarchical organization of mitochondrial functions. *Cell Rep.* **19**, 2557 (2017). 28636943 10.1016/j.celrep.2017.05.073

[34] Zhang, H. et al. Exosome-mediated targeted delivery of miR-210 for angiogenic therapy after cerebral ischemia in mice. *J. Nanobiotechnol.* **17**, 29 (2019). 10.1186/s12951-019-0461-7 PMC6379944 30782171

[35] Yang, W. et al. Exosomes from young healthy human plasma promote functional recovery from intracerebral hemorrhage via counteracting ferroptotic injury. *Bioact. Mater.* **27**, 1 (2023). 37006825 10.1016/j.bioactmat.2023.03.007 PMC10060149

[36] Luo, Z. et al. Human bone marrow mesenchymal stem cell-derived extracellular vesicles inhibit shoulder stiffness via let-7a/Tgfbr1 axis. *Bioact. Mater.* **17**, 344 (2022). 35386460 10.1016/j.bioactmat.2022.01.016 PMC8965035

[37] Palamà, M. E. F. et al. Xeno-free cultured mesenchymal stromal cells release extracellular vesicles with a “therapeutic” miRNA cargo ameliorating cartilage inflammation in vitro. *Theranostics* **13**, 1470 (2023). 37056573 10.7150/thno.77597 PMC10086204

[38] Poggio, M. et al. Suppression of exosomal PD-L1 induces systemic anti-tumor immunity and memory. *Cell* **177**, 414 (2019). 30951669 10.1016/j.cell.2019.02.016 PMC6499401

[39] Rojas, C. et al. A novel and potent brain penetrant inhibitor of extracellular vesicle release. *Br. J. Pharmacol.* **176**, 3857 (2019). 31273753 10.1111/bph.14789 PMC6780992

[40] Xie, Y. et al. Astrocyte-neuron crosstalk through Hedgehog signaling mediates cortical synapse development. *Cell Rep.* **38**, 110416 (2022). 35196485 10.1016/j.celrep.2022.110416 PMC8962654

[41] Perea, G., Navarrete, M. & Araque, A. Tripartite synapses: astrocytes process and control synaptic information. *Trends Neurosci.* **32**, 421 (2009). 19615761 10.1016/j.tins.2009.05.001

[42] Chen, L. et al. Tailored reconstituted lipoprotein for site-specific and mitochondria-targeted cyclosporine a delivery to treat traumatic brain injury. *ACS Nano* **14**, 6636 (2020). 32464051 10.1021/acsnano.9b09186

[43] Lamade, A. M. et al. Aiming for the target: mitochondrial drug delivery in traumatic brain injury. *Neuropharmacology* **145**, 209 (2019). 30009835 10.1016/j.neuropharm.2018.07.014 PMC6309489

[44] Frezza, C. et al. OPA1 controls apoptotic cristae remodeling independently from mitochondrial fusion. *Cell* **126**, 177 (2006). 16839885 10.1016/j.cell.2006.06.025

[45] Lai, Y. et al. Restoration of L-OPA1 alleviates acute ischemic stroke injury in rats via inhibiting neuronal apoptosis and preserving mitochondrial function. *Redox Biol.* **34**, 101503 (2020). 32199783 10.1016/j.redox.2020.101503 PMC7327985

[46] Lu, H. et al. miRNA-382-5p carried by extracellular vesicles in osteoarthritis reduces cell viability and proliferation, and promotes cell apoptosis by targeting PTEN. *DNA Cell Biol.* **41**, 1012 (2022). 36413378 10.1089/dna.2021.0726

[47] Chen, Y. & Wang, X. miRDB: an online database for prediction of functional microRNA targets. *Nucleic Acids Res.* **48**, D127 (2020). 31504780 10.1093/nar/gkz757 PMC6943051

[48] Agarwal, V., Bell, G. W., Nam, J. W. & Bartel, D. P. Predicting effective microRNA target sites in mammalian mRNAs. *Elife* **4**, e05005 (2015). 26267216 10.7554/eLife.05005 PMC4532895

[49] Krüger, J. & Rehmsmeier, M. RNAhybrid: microRNA target prediction easy, fast and flexible. *Nucleic Acids Res.* **34**, W451 (2006). 16845047 10.1093/nar/gkl243 PMC1538877

[50] Human genomics. The Genotype-Tissue Expression (GTEx) pilot analysis: multitissue gene regulation in humans. *Science* **348**, 648 (2015). 25954001 10.1126/science.1262110 PMC4547484

[51] Zakarya, R. et al. Nitroxides affect neurological deficits and lesion size induced by a rat model of traumatic brain injury. *Nitric Oxide* **97**, 57 (2020). 32061903 10.1016/j.niox.2020.02.001

[52] Yang, L. et al. Extracellular vesicle-mediated delivery of circular RNA SCMH1 promotes functional recovery in rodent and nonhuman primate ischemic stroke models. *Circulation* **142**, 556 (2020). 32441115 10.1161/CIRCULATIONAHA.120.045765

[53] Yu, X. et al. Extracellular vesicle-mediated delivery of circDYM alleviates CUS-induced depressive-like behaviours. *J. Extracell. Vesicles* **11**, e12185 (2022). 35029057 10.1002/jev2.12185 PMC8758833

[54] Kang, J. Y. et al. Engineered small extracellular vesicle-mediated NOX4 siRNA delivery for targeted therapy of cardiac hypertrophy. *J. Extracell. Vesicles* **12**, e12371 (2023). 37795828 10.1002/jev2.12371 PMC10552075

[55] Alvarez-Erviti, L. et al. Delivery of siRNA to the mouse brain by systemic injection of targeted exosomes. *Nat. Biotechnol.* **29**, 341 (2011). 21423189 10.1038/nbt.1807

[56] Revill, G., Carlisi, C., David, A., Poole, N. & Bell, V. Towards further progress in traumatic brain injury. *Lancet Neurol.* **22**, 109 (2023). 36681440 10.1016/S1474-4422(22)00527-0

[57] Meyfroidt, G. et al. Management of moderate to severe traumatic brain injury: an update for the intensivist. *Intensive Care Med.* **48**, 649 (2022). 35595999 10.1007/s00134-022-06702-4

[58] Nekludov, M., Mobarrez, F., Gryth, D., Bellander, B. M. & Wallen, H. Formation of microparticles in the injured brain of patients with severe isolated traumatic brain injury. *J. Neurotrauma* **31**, 1927 (2014). 24956150 10.1089/neu.2013.3168

[59] Dickens, A. M. et al. Astrocyte-shed extracellular vesicles regulate the peripheral leukocyte response to inflammatory brain lesions. *Sci. Signal.* **10**, eaai7696 (2017). 28377412 10.1126/scisignal.aai7696 PMC5590230

[60] Wang, J., Wang, J., Li, X. & Shu, K. Cell-derived exosomes as therapeutic strategies and exosome-derived microRNAs as biomarkers for traumatic brain injury. *J. Clin. Med.* **11**, 3223 (2022). 35683610 10.3390/jcm11113223 PMC9181755

[61] Long, X. et al. Astrocyte-derived exosomes enriched with miR-873a-5p inhibit neuroinflammation via microglia phenotype modulation after traumatic brain injury. *J. Neuroinflammation* **17**, 89 (2020). 32192523 10.1186/s12974-020-01761-0 PMC7082961

[62] Wu, X. et al. Astrocyte-derived extracellular vesicular miR-143-3p dampens autophagic degradation of endothelial adhesion molecules and promotes neutrophil transendothelial migration after acute brain injury. *Adv. Sci. (Weinh.)* **11**, e2305339 (2023). 38044319 10.1002/advs.202305339 PMC10837358

[63] Lv, B., Liu, X., Zhu, X. & Huang, M. miR-382-5p promotes cell invasion in hepatocellular carcinoma by targeting PTEN to activate PI3K/Akt signaling pathway. *World J. Surg. Oncol.* **20**, 175 (2022). 35655254 10.1186/s12957-022-02638-7 PMC9161500

[64] Wu, H. et al. MicroRNA-382-5p inhibits osteosarcoma development and progression by negatively regulating VEZF1 expression. *Oncol. Lett.* **22**, 752 (2021). 34539856 10.3892/ol.2021.13013 PMC8436354

[65] Zhang, L., Zhu, H., Teng, X., Sheng, X. & Yu, B. Modulation of miR-382-5p reduces apoptosis of myocardial cells after acute myocardial infarction. *Autoimmunity* **54**, 195 (2021). 34042547 10.1080/08916934.2021.1910812

[66] Bennett, C. F., Latorre-Muro, P. & Puigserver, P. Mechanisms of mitochondrial respiratory adaptation. *Nat. Rev. Mol. Cell Biol.* **23**, 817 (2022). 35804199 10.1038/s41580-022-00506-6 PMC9926497

[67] Giacomello, M., Pyakurel, A., Glytsou, C. & Scorrano, L. The cell biology of mitochondrial membrane dynamics. *Nat. Rev. Mol. Cell Biol.* **21**, 204 (2020). 32071438 10.1038/s41580-020-0210-7

[68] Sies, H. et al. Defining roles of specific reactive oxygen species (ROS) in cell biology and physiology. *Nat. Rev. Mol. Cell Biol.* **23**, 499 (2022). 35190722 10.1038/s41580-022-00456-z

[69] High, K. A. & Roncarolo, M. G. Gene therapy. *N. Engl. J. Med.* **381**, 455 (2019). 31365802 10.1056/NEJMra1706910

[70] Singh, R. R., Mondal, I., Janjua, T., Popat, A. & Kulshreshtha, R. Engineered smart materials for RNA based molecular therapy to treat Glioblastoma. *Bioact. Mater.* **33**, 396 (2024). 38059120 10.1016/j.bioactmat.2023.11.007 PMC10696434

[71] Schulz-Siegmund, M. & Aigner, A. Nucleic acid delivery with extracellular vesicles. *Adv. Drug Deliv. Rev.* **173**, 89 (2021). 33746014 10.1016/j.addr.2021.03.005

[72] Pineda, J. A., Wang, K. K. & Hayes, R. L. Biomarkers of proteolytic damage following traumatic brain injury. *Brain Pathol.* **14**, 202 (2004). 15193033 10.1111/j.1750-3639.2004.tb00054.x PMC8095934

