# Supplementary material for: Astrocyte–neuron crosstalk through extracellular vesicle-shuttled miRNA-382-5p promotes traumatic brain injury
Source: Exp Mol Med. 2024 Dec 2;56(12):2642–58. doi: 10.1038/s12276-024-01355-3 (PMC11671591; doi:10.1038/s12276-024-01355-3)
Supplement: Supplementary file 1 — Supplementary Information [file 12276_2024_1355_MOESM1_ESM.pdf]

Supplementary Materials for

Astrocyte–neuron crosstalk through extracellular vesicle-shuttled miRNA-382-5p promotes traumatic brain injury

This PDF file includes:

- Supplementary Table 1 to 5.. 2
- Supplementary Fig. 1 to 10 8

- **Supplementary Table 1 to 5**

Supplementary Table 1 Demographic and Clinical Characteristics of TBI and Control group

Characteristic	Category	Control n (%)	TBI n (%)	p-value
Age	Mean±SD	54.3±17.9	54.8±15.3	0.856
Gender	Female	24(40.0%)	19(31.7%)	0.341
	Male	36(60.0%)	41(68.3%)	

Supplementary Table 2 Demographic and clinical characteristics of TBI patients with good and poor outcomes

Characteristic	Category	TBI^{gp} n (%)	TBI^{pp} n (%)	p-value
Age	Mean ± SD	47.5 ± 13.9	62.2 ± 13.0	**< 0.001**
Gender				0.78
	Female	9(30.0%)	10(33.3%)	
	Male	21(70.0%)	20(66.7%)	
GCS				**0.038**
	≥9	20(66.7%)	12(40.0%)	
	< 9	10(33.3%)	18(60.0%)	
Injury mechanism				0.770
	Accident-1	10(33.3%)	13(43.3%)	
	Fall-2	15(50.0%)	12(40.0%)	
	Assault-3	3(10.0%)	2(6.7%)	
	Other-4	2(6.7%)	3(10.0%)	
Marshall's scale				**< 0.001**
	Class I/II	18(60.0%)	4(13.3%)	
	Class III/IV	10(33.3%)	17(56.7%)	
	Class V/VI	2(6.7%)	9(30.0%)	
ISS				**0.015**
	Mild to moderate (1-8)	6(20.0%)	2(6.7%)	
	Serious (9-15)	15(50.0%)	11(36.7%)	

Supplementary Table 2 (Continued) Demographic and clinical characteristics of TBI patients with good and poor outcomes

Characteristic	Category	TBI^{gp} n (%)	TBI^{pp} n (%)	p-value
	Serious (16-24)	9(30.0%)	15(50.0%)	
	Critical (25-75)	0(0.0%)	2(6.7%)	
tSAH				**0.002**
	No	23(76.7%)	11(36.7%)	
	Yes	7(23.3%)	19(63.3%)	
SDH				0.108
	No	22(73.3%)	16(53.3%)	
	Yes	8(26.7%)	14(46.7%)	
EDH				0.426
	No	20(66.7%)	17(56.7%)	
	Yes	10(33.3%)	13(43.3%)	
WBC	Mean ± SD	14.09 ± 6.33	16.21 ± 5.88	0.185
Glucose	IQ range	9.6[6.90,15.29]	9.63[7.52,13.66]	0.842
D-dimer	Mean ± SD	24.11 ± 18.59	27.62 ± 20.15	0.486
miR-382-5p	IQ range	1.54[1.37,2.20]	2.30[1.78,2.78]	**< 0.001**

TBI^{gp}, TBI patients with good prognosis; TBI^{pp}, TBI patients with poor prognosis; GCS, glasgow coma scale; ISS, injury severity score; tSAH, traumatic subarachnoid hemorrhage; SDH, subdural hemorrhage; EDH, epidural hemorrhage; WBC, white blood cell.

Supplementary Table 3 The miR-382-5p level was used to predict the functional outcome of TBI patients after 6 months in the multivariate logistic regression model

Predictor	β	OR (95%CI)	P-value
Age	0.128	1.136(1.031-1.253)	**0.010**
GCS	-2.180	0.113(0.013-0.982)	**0.048**
Marshall's scale: Class III/IV (refer to Class I/II)	2.526	12.508(1.095-142.927)	**0.042**
Marshall's scale: Class V/VI (refer to Class I/II)	0.988	2.686(0.170-42.419)	0.483
ISS	0.003	1.003(0.882-1.140)	0.968
tSAH	1.461	4.312(0.637-29.178)	0.134
miR-382-5p	2.759	15.783(2.152-115.769)	**0.007**

GCS, glasgow coma scale; ISS, injury severity score; tSAH, traumatic subarachnoid hemorrhage.

Supplementary Table 4 MiRNAs' sequences used in this study.

Gene	Sequence (5'-3')
cel-miR-39	UCACCGGGUGUAAAUCAGCUUG
miR-382-5p mimics	GAAGUUGUUCGUGGUGGAUUCG
miR-382-5p mimics NC	UUGUACUACACAAAAGUACUG
miR-382-5p inhibitor	CGAAUCCACCACGAACAACUUC
miR-382-5p inhibitor NC	CAGUACUUUUGUGUAGUACAA

Supplementary Table 5 Primers' sequences used in this study.

PCR primers	Forward (5'-3')	Reverse (5'-3')
miR-382-5p (human/mouse)	AAGTTGTTCGTGGTGGATTCG	Provided in the kit
miR-361-5p (human/mouse)	TTATCAGAATCTCCAGGGGTAC	
miR-224-5p (human/mouse)	GTCACTAGTGGTTCCGTTTAG	
miR-654-5p (human/mouse)	TATGTCTGCTGACCATCACCTT	
miR-39 (C.elegans)	ACCGGGTGTAAATCAGCTTG	
U6 (mouse)	GGAACGATACAGAGAAGATTAG C	TGGAACGCTTCA CGAATTTGCG
Pre-miR-382 (mouse)	ACTTGAAGAGAAGTTGTTCGTG	AAGTGTTGTCCGT GAATGATTCG
β-actin (mouse)	CATCCGTAAAGACCTCTATGCCA AC	ATGGAGCCACCG ATCCACA

- **Supplementary Fig. 1 to 10**

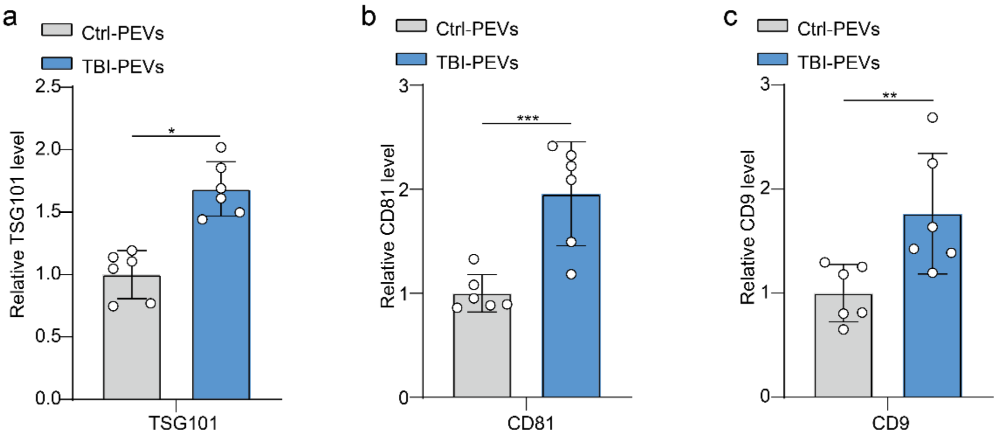

Supplementary Fig. 1 Histogram quantization the presence of EVs-specific markers between TBI patients and healthy controls.

a-c Densitometric quantifications of the EVs markers shown are normalized to the plasma volume (n=6 per group; Student' s t-test). Data represent mean ± SD; **p* < 0.05, ***p* < 0.01, ****p* < 0.001.

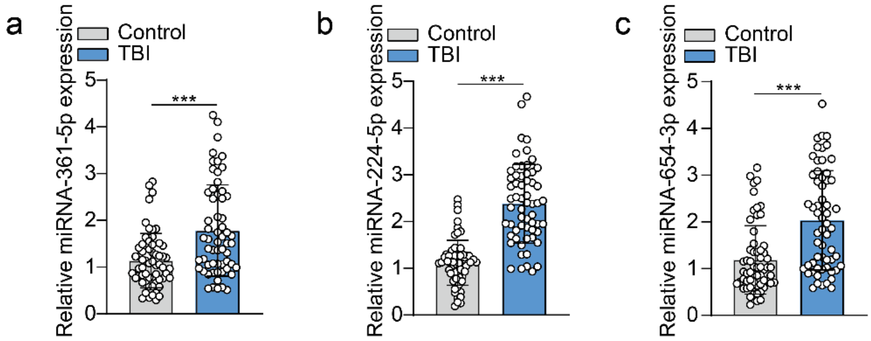

Supplementary Fig. 2 Quantitative real-time PCR analysis verify the differently expressed EVs-miRNAs between TBI patients and healthy controls.

a-c Related fold changes of the mRNA levels of miRNA-361-5p, miRNA-224-5p and miRNA-654-3p in plasma EVs between TBI patients and healthy controls (n=60 per group; Student' s t-test). Data represent mean ± SD; ****p* < 0.001, *NS*: not significant.

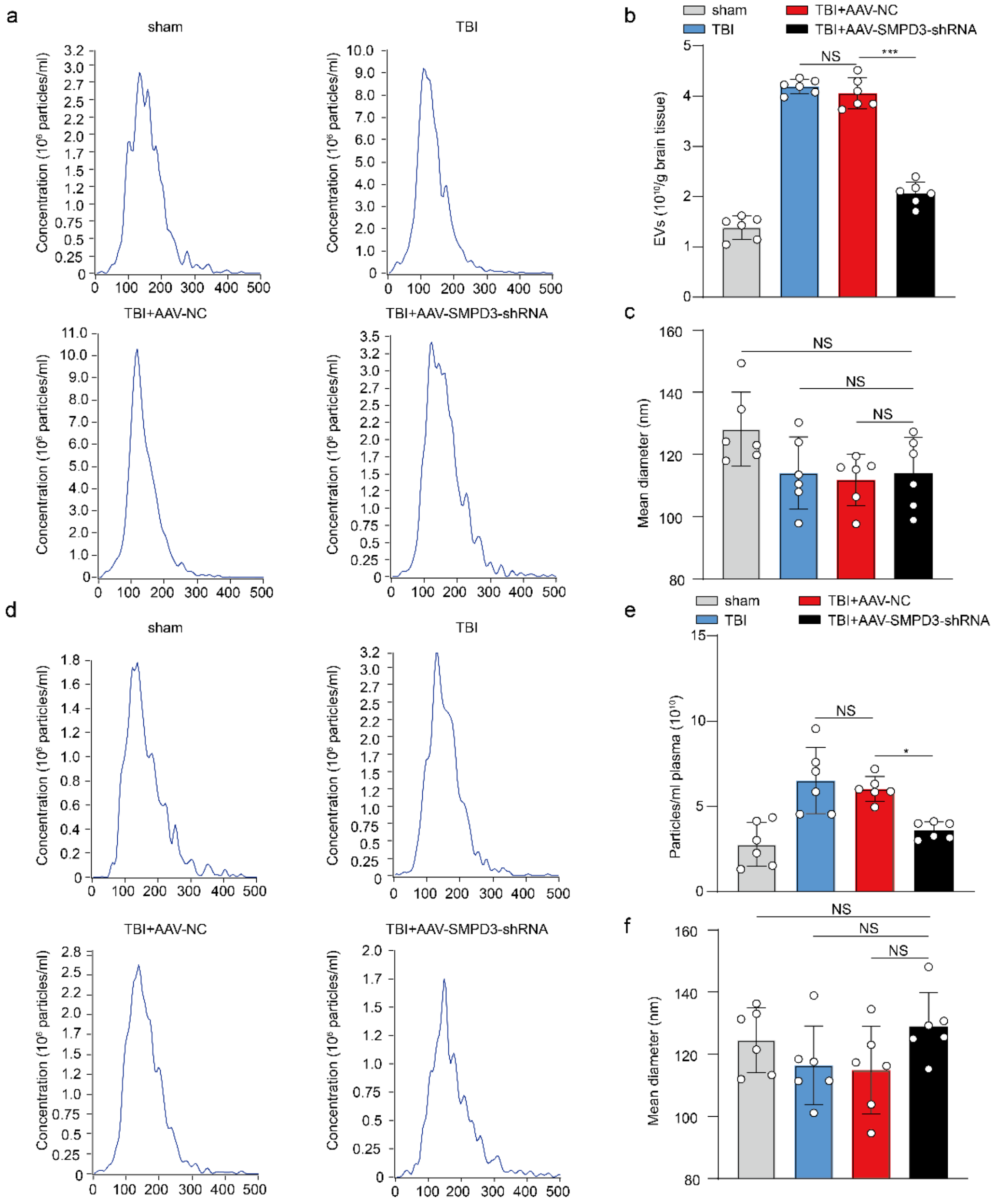

Supplementary Fig. 3 The quality of EVs in brain and plasma tissue of mice with TBI after AAV-SMPD3-shRNA virus infection.

a The size distribution and number of particles per milliliter using samples isolated from brain-derived EVs are shown. **b,c** The concentration and size of brain-derived EVs isolated under the experimental conditions are shown (n=6 per group; Student' s t-test). **d** The size distribution and number of particles per milliliter using samples isolated from plasma-derived EVs are shown. **e,f** The concentration and size of plasma-derived EVs isolated under the experimental conditions are shown (n=6 per group; Student' s t-test). Data represent mean ± SD; **p* < 0.05, ****p* < 0.001, *NS*: not significant.

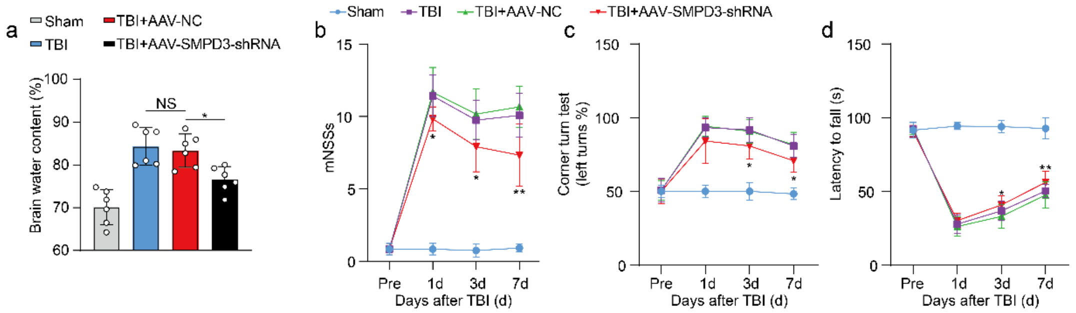

Supplementary Fig. 4 The AAV-SMPD3-shRNA can ameliorate brain edema and neurological dysfunction in the TBI mouse model.

a Statistical analysis of brain edema in the AAV-SMPD3-shRNA group or AAV-SMPD3-NC group (n=6 per group, one-way ANOVA). **b-d** Neurological function deficit scores in the AAV-SMPD3-shRNA group or AAV-SMPD3-NC group (n=12 per group, two-way ANOVA). Data represent mean ± SD; *$p < 0.05$, **$p < 0.01$, *NS*: not significant.

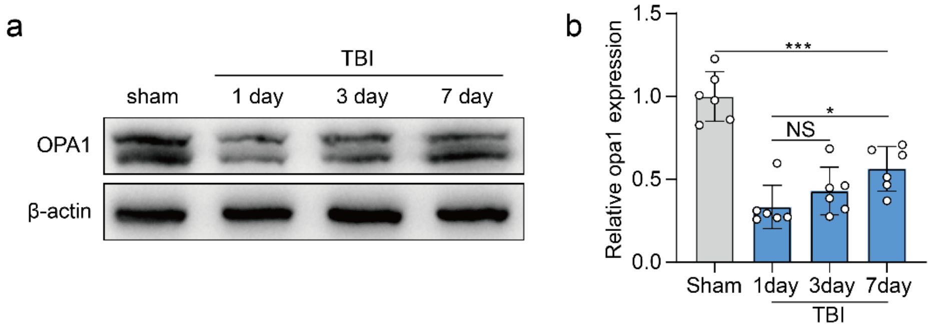

Supplementary Fig. 5 The expression of OPA1 is decreased in the perilesional cortex of TBI mice.

a,b WB analysis and densitometric quantification of OPA1 protein in the perilesional cortex of TBI mice (n=6 per group, one-way ANOVA). Data represent mean ± SD; *$p < 0.05$, ***$p < 0.001$, *NS*: not significant.

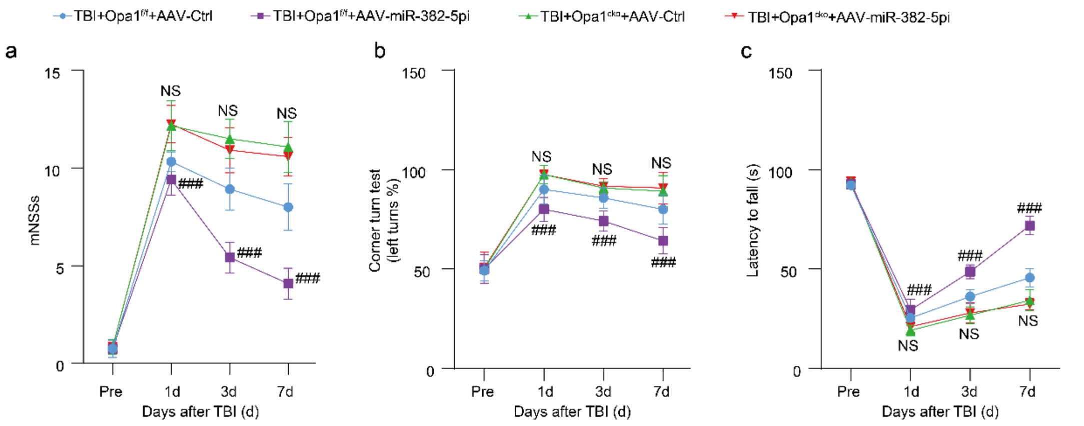

Supplementary Fig. 6 The AAV-miR-382-5pi did not exhibit therapeutic benefits in alleviating neurological dysfunction following TBI in *Opa1*^{CKO} mice.

a-c Neurological function deficit scores in the AAV-miR-382-5pi group or AAV-Ctrl group after TBI in *Opa1*^{CKO} mice (n=12 per group, two-way ANOVA). Data represent mean ± SD; ###$p < 0.001$ vs. TBI + *Opa1*$^{f/f}$ + AAV-miR-382-5pi; *NS*: not significant, vs. TBI + *Opa1*^{cko} + AAV-Ctrl.

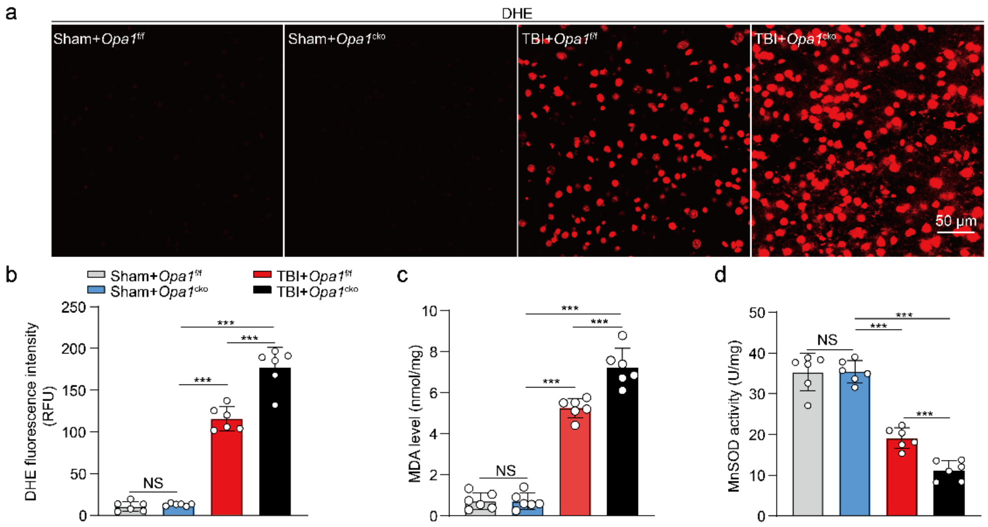

Supplementary Fig. 7 Neuron-specific OPA1 conditional gene knockout aggravates oxidative stress after TBI.

a,b Typical images of DHE staining and statistical analysis of DHE fluorescence intensities of the perilesional cortex 24 h after TBI (n=6 per group, one-way ANOVA), which indicates ROS levels. **c,d** Effects of OPA1 knockout on MDA levels and MnSOD activities (n=6 per group, one-way ANOVA). Data represent mean ± SD; ***$p < 0.001$, *NS*: not significant.

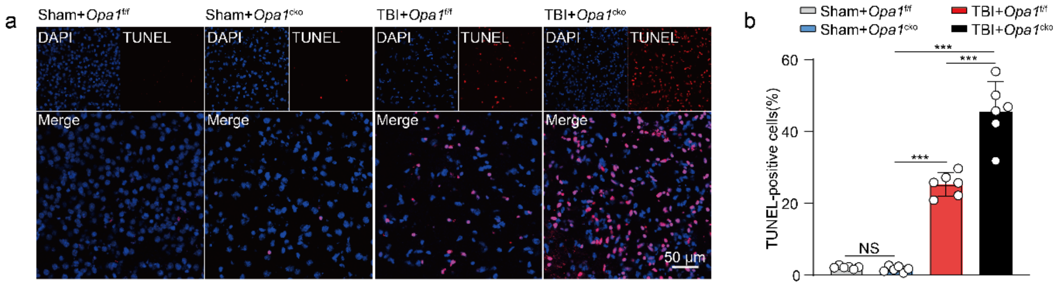

Supplementary Fig. 8 Neuron-specific OPA1 conditional gene knockout aggravates apoptosis after TBI.

a,b Representative TUNEL staining images and quantitative analyses of TUNEL+ cells in the *Opa1*$^{f/f}$ group and *Opa1*^{CKO} group (n=6 per group, one-way ANOVA). Data represent mean ± SD; ***$p < 0.001$, *NS*: not significant.

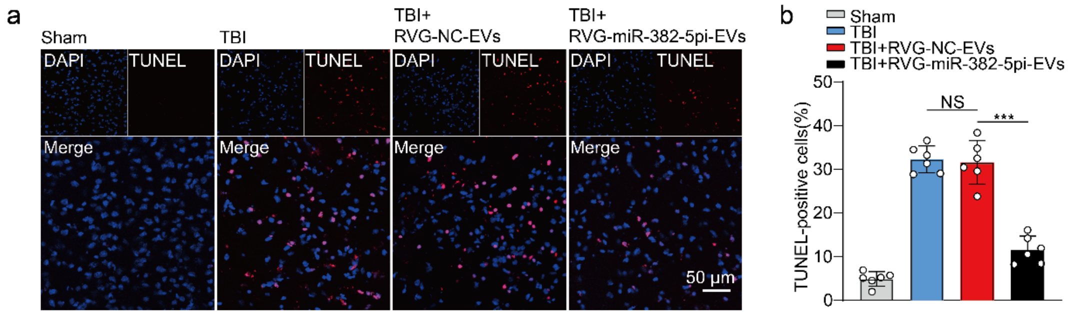

Supplementary Fig. 9 RVG-miR-382-5pi-EVs reduces apoptosis after TBI.

a,b Representative TUNEL staining images and quantitative analyses of $TUNEL^+$ cells injected with or without RVG-miR-382-5pi-EVs (n=6 per group, one-way ANOVA). Data represent mean ± SD; ****p* < 0.001, *NS*: not significant.

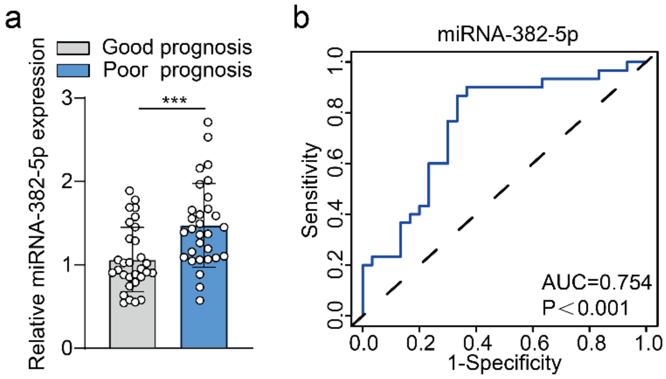

Supplementary Fig. 10 EV-miRNA-382-5p effectively predict the outcome of TBI.

a Related fold changes of the mRNA levels of miRNA-382-5p in plasma EVs of patients with good TBI prognosis (GOS 4 to 5) and patients with poor TBI prognosis (GOS 1 to 3) (n=30 per group; Student' s t-test). **b** ROC curve for individual miRNA-382-5p in plasma EVs to separate good (GOS 4 to 5) (n=30) outcomes from poor (GOS 1 to 3) (n=30) outcomes 6 months later. Data represent mean ± SD; ***$p <$ 0.001.
